# Assessing the function of the alternative electron transport chain in the *Cryptosporidium parvum* mitosome

**DOI:** 10.1128/mbio.01120-25

**Published:** 2025-11-13

**Authors:** Silu Deng, Wandy Beatty, L. David Sibley

**Affiliations:** 1 Department of Molecular Microbiology, Washington University School of Medicine 12275 , St. Louis, Missouri, USA; Rutgers-New Jersey Medical School, Newark, New Jersey, USA

**Keywords:** cryptosporidiosis, alternative oxidase, type II NADH dehydrogenase, mitochondria

## Abstract

*Cryptosporidium parvum and C. hominis* possess a remanent mitochondrion called the mitosome, which lacks DNA, the tricarboxylic acid cycle, a conventional electron transport chain, and ATP synthesis. The mitosome retains ubiquinone and iron-sulfur cluster biosynthesis pathways, which require protein import that relies on the membrane potential. It was previously proposed that the membrane potential may be generated by a transhydrogenase (TH) that pumps protons out of the mitosome. This pathway was also proposed to rely on an alternative oxidase (AOX) and type II NADH dehydrogenase (NDH2), which also exists in plants, some fungi, and several protozoan parasites. To examine this model, we determined the location and function of AOX and NDH2 in *C. parvum*. Surprisingly, we observed that NDH2 was localized to parasite surface membranes instead of the mitosome. Furthermore, a ∆*ndh2* knockout (KO) strain was readily obtained, indicating that this protein is not essential for parasite growth. Although AOX exhibited a mitosome-like staining pattern, we readily obtained a ∆*aox* KO strain, which did not exhibit a difference in MitoTracker, indicating that AOX is likely not involved in membrane potential and is dispensable for parasite growth. The growth of the ∆*aox* strain was inhibited by the AOX inhibitors, salicylhydroxamic acid and 8-hydroxyquinoline, to the same extent as wild type, indicating that AOX is not the target of these inhibitors in *C. parvum*. Collectively, our studies indicate that NDH2 and AOX are non-essential genes in *C. parvum*, supporting an alternative mechanism for maintaining the mitosome membrane potential.

**IMPORTANCE:**

Cryptosporidiosis is a leading cause of diarrhea in young children and immunocompromised individuals, particularly AIDS/HIV patients. The only FDA-approved drug against cryptosporidiosis, nitazoxanide, has limited effectiveness in immunocompromised patients and is not approved for use in children under 1 year. Genomic analysis and previous studies proposed an alternative respiration pathway involving alternative oxidase (AOX) and type II NAD(P)H dehydrogenase (NDH2), which are thought to generate the mitosome membrane potential in *Cryptosporidium parvum*. Additionally, AOX was nominated as potential drug targets, based on its absence in mammalian hosts and sensitivity of parasite growth to known inhibitors of AOX. However, our study demonstrated that NDH2 is not localized in the mitosome, AOX is non-essential for parasite growth, and knockout lines lacking this enzyme are equally sensitive to AOX inhibitors. These findings indicate that AOX and NDH2 are not ideal candidates for future drug development against cryptosporidiosis and force a re-evaluation of models of how the mitosome generates its membrane potential.

## INTRODUCTION

Mitochondria originated from α-proteobacterial endosymbionts and serve as ATP-generating factories in aerobic eukaryotes (1). During evolution, their genomes and proteomes have radically evolved, affecting biological processes and structure. Despite the trend toward increased genome complexity, many parasitic and symbiotic organisms have reduced mitochondrion-related organelles (2, 3). The reduction primarily manifests in loss of the mitochondrial genome, the simplified structure, decreased size, and specialized or diminished functionality, which often makes it difficult to recognize and identify these relict mitochondrial compartments. The function of mitochondrion-related organelles ranges widely across different organisms, including hydrogenosomes comprising anaerobic energy metabolism and the mitosomes retaining the Fe-S biosynthesis with a loss of energy-generating machinery (4).

Apicomplexan parasites vary considerably in the composition and retention of pathways that are typical of mitochondria, including components of the electron transport chain (ETC), tricarboxylic acid (TCA) cycle, and complex for ATP synthesis (5). Mitochondria in both *Plasmodium* spp. and *T. gondii* encode a complete electron transport chain (ETC), TCA cycle, and complex V for ATP synthesis similar to that of most other eukaryotes (6, 7). However, they differ from mammalian cells by the presence of an alternative complex I, type II NADH dehydrogenase (NDH2), replacing the canonical complex I in the ETC (8). In contrast, the genus *Cryptosporidium* possesses mitochondrial relicts that are much smaller in size and have reduced functionality (9, 10). The gastric-dwelling *Cryptosporidium* species, including *C. muris* and *C. andersonii*, likely contain a functional ETC and complete TCA cycle for energy metabolism as orthologs of these enzymes are encoded in the genome (4, 6, 10). In contrast, the intestine-dwelling species, particularly *Cryptosporidium parvum* and *Cryptosporidium hominis*, lack the TCA cycle and retain only two subunits of the ATPase and are thus incapable of generating ATP. Instead, it has been proposed that they have an alternative ETC consisting of two dehydrogenases, NDH2 and malate-quinone oxidoreductase (MQO), as well as an alternative oxidase (AOX) (4, 10). This remnant organelle is referred to as the mitosome, which is a roughly spherical, 150–-300 nm in diameter, double membrane-bounded organelle between the nucleus and crystalloid body at the posterior end of the sporozoites (11).

NDH2 proteins have been widely described in plants, fungi, and protists but are absent in mammalian mitochondria (12). Many plants, fungi, and protozoa possess both complex I and NDH2, while apicomplexan parasites only retain NDH2 (13). In the proposed modified ETC of *C. parvum*, NDH2 catalyzes the oxidation of NADH to NAD^+^, followed by the reduction of quinone to quinol, resulting in electron transfer from NADH to quinone (14). However, unlike the canonic complex I, which contains multiple subunits that are involved in electron transfer and proton pumping, NDH2 is embedded in the inner mitochondrial membrane where it transfers electrons to CoQ, but does not mediate proton pumping (14, 15). Due to their absence in mammals, type II NADH dehydrogenases have been proposed as attractive drug targets against *Mycobacterium tuberculosis* (16) and as potential drug targets against apicomplexan parasites (17). MQO is another possible electron donor for the mitosome ETC encoded in *C. parvum*. MQO catalyzes the reversible NAD^+^-independent oxidation of malate to oxaloacetate and mediates electron transport through the reduction of quinone in mitochondria, which has been reported to be a pH-dependent process based on functional studies *in vitro* (18, 19). The only electron acceptor identified from the genome of *C. parvum* is AOX, a cyanide-resistant ubiquinol oxidase found in the mitochondria of all plants as well as some fungi and protozoa, which can accept electrons directly from coenzyme Q and catalyze the reduction of oxygen to water (20, 21). AOX is not encoded in the genomes of either *T. gondii* or *Plasmodium* spp. (7), while it plays a crucial role in respiration and development of both bloodstream and procyclic forms of *Trypanosoma brucei*, making it a viable chemotherapeutic target for African trypanosomiasis (22). A previous study has reported that the AOX inhibitors, salicylhydroxamic acid (SHAM) and 8-hydroxyquinoline (8-HQ), inhibit the growth of *C. parvum* in cell culture (23, 24), leading to the suggestion that AOX could be a potential drug target for cryptosporidiosis. Membrane-bound NAD(P) transhydrogenase (TH) facilitates the reversible transfer of hydride ions between NAD(H) and NADP(H) while translocating protons across the mitochondrial inner membrane, an activity that is conserved across prokaryotes and eukaryotes (25). In mammals, TH proteins reside in the mitochondria inner membrane (26), whereas *Plasmodium* TH is found in the apicoplast rather than mitochondria (27).

It was previously proposed that TH may contribute to the mitosome membrane potential in *C. parvum* (11, 28). Alternatively, it was previously suggested based on analysis of the genome content of *C. parvum* and *C. hominis* that NDH2 and AOX may participate in an alternative ETC that contributes to the mitosome membrane potential (7, 10). However, no studies have investigated the localization and essentiality of these components. In the present study, we localized the AOX and NDH2 proteins using epitope tags and tested their essentiality for growth. Our findings indicate that NDH2 is not localized in the mitosome, and neither NDH2 nor AOX is essential for growth of *C. parvum*, forcing a revision of current models for how the mitosome membrane potential is generated.

## RESULTS

### Proposed model for ETC in mitosome of *C. parvum*

**Fig 1:**
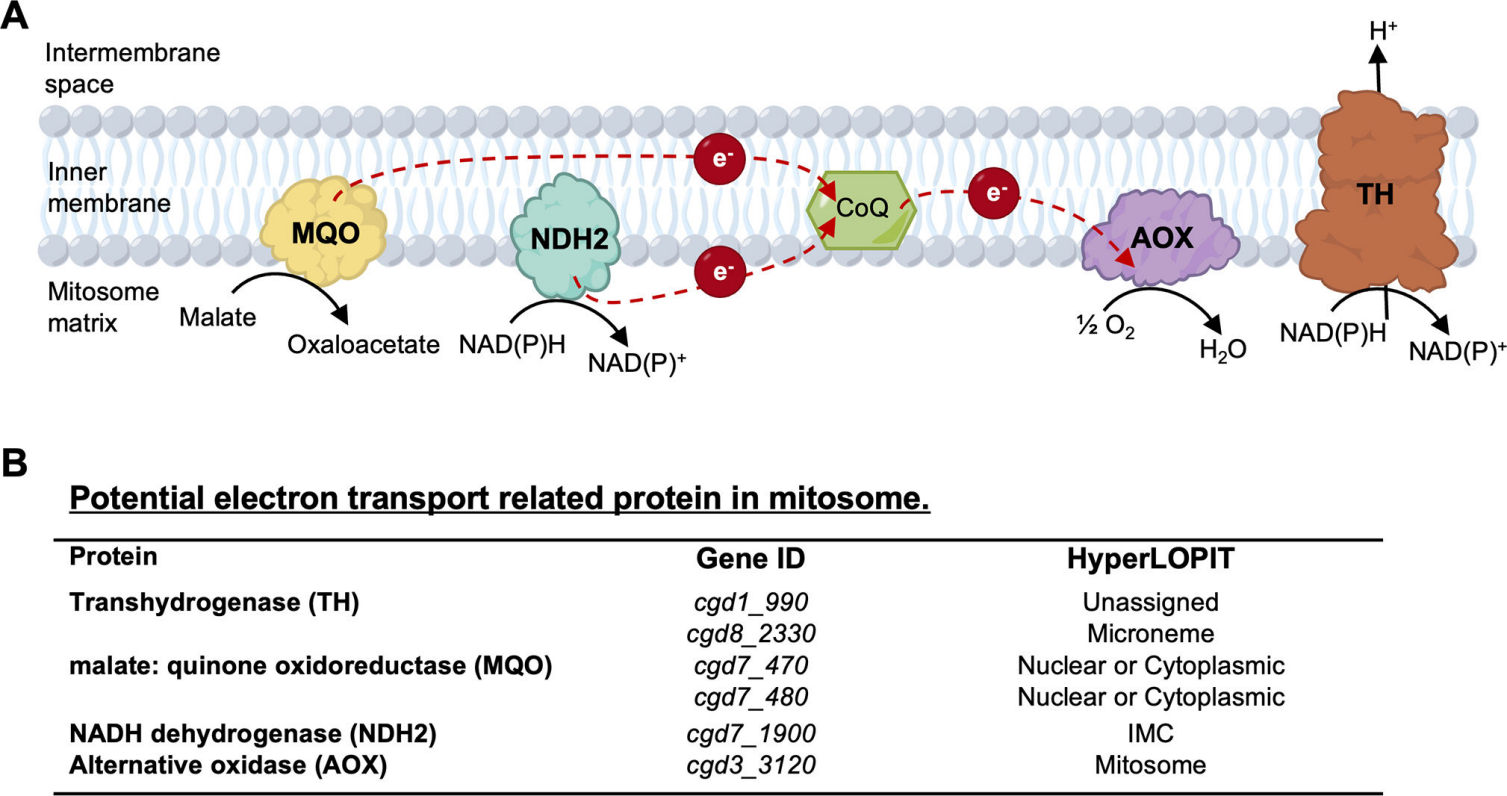
Proposed model for ETC in *C. parvum* mitosome. (**A**) Schematic diagram of the proposed model for ETC in *C. parvum* mitosome. MQO, malate: quinone oxidoreductase; NDH2, type II NAD(P)H dehydrogenase; CoQ, coenzyme Q; AOX, alternative oxidase; TH, transhydrogenase. (**B**) Summary of annotated genes from genomic and proteomic databases for *C. parvum*. Gene IDs were obtained from the NCBI gene database (https://www.ncbi.nlm.nih.gov/gene). Predicted localization was based on the HyperLOPIT proteomic data set (29).

Genomic analysis of *C. parvum* and *C. hominis* revealed a progressive reduction in mitochondrial functions in these species that infect the small intestine relative to *C. muris* which resides in the stomach (6, 7, 10). Although *C. parvum* retains genes encoding the proteins involved in the ubiquinone biosynthesis, only a few enzymes mediating the ETC were identified in *C. parvum*, including MQO, NDH2, and AOX (4, 6, 10). Thus, a simplified model for an alternative ETC in *C. parvum* mitosome was proposed in previous reviews based on comparative genomics (4). In this model, electrons are produced during oxidation of malate to oxaloacetate by MQO, or dehydrogenation of NAD(P)H to NAD(P)^+^ by NDH2, and then transferred to CoQ, which releases the electrons to AOX via the reduction of quinone to quinol (Fig. 1A). AOX subsequently catalyzes the oxidation of quinol and the reduction of oxygen to water, and this alternative ETC is coupled to proton pumping by TH (Fig. 1A). Two MQO-like proteins (encoded by *cgd7_470* and *cgd7_480*), two TH-like proteins (encoded by *cgd1_990* and *cgd8_2330*), one NDH2 (encoded by *cgd7_1900*), and one AOX (encoded by *cgd3_3120*) are identified from the genomic analysis of *C. parvum* (10, 25). The HyperLOPIT proteomic database (29) indicated a nuclear/cytoplasmic location for both MQOs, microneme location for TH (*cgd8_2330*), and unassigned for TH (*cgd1_990*), while NDH2 was found with the inner membrane complex (IMC) and AOX fractionated with the mitosome, respectively (Fig. 1B). Here, we primarily focused on characterizing the phylogeny, localization, and function of NDH2 and AOX in *C. parvum*.

To better understand the evolutionary conservation of CpAOX and CpNDH2, we compared their sequences with orthologs from a range of organisms, including algae, fungi, plants, bacteria, and other protozoa. Maximum-likelihood phylogenetic analysis revealed that CpAOX clusters closely with orthologs from other *Cryptosporidium* species. Although more distantly related to AOX orthologs from other protozoa, fungi, and algae, CpAOX consistently groups within the canonical AOX lineage rather than with other terminal oxidases such as the plastid terminal oxidase (Fig. S1A). Sequence alignment further showed that CpAOX contains a highly conserved region including the characteristic di-iron center (Fig. S1B). CpNDH2 falls within the same clade as orthologs from *T. gondii* and *Plasmodium spp*. (Fig. S2A). While CpNDH2 displays relatively low sequence conservation across its entire length, it retains key conserved functional residues such as those involved in FAD stabilization and quinone binding within the first Rossmann domain, residues responsible for NAD(P)H binding in the second Rossmann domain, and conserved residues in the C-terminal region (Fig. S2B) (30). To further verify the identities of CpAOX and CpNDH2 in *C. parvum*, we performed BLASTP searches against the nr database and compiled the top hits in other organisms. These searches returned hits with high expect values that were annotated as AOX and NDH2, respectively (Table S1). Reciprocal BLAST of these hits against the protein database of *C. parvum* confirmed that CpAOX and CpNDH2 are the only matching sequences, supporting their identities as originally annotated (Table S1).

### CpNDH2 is present at the parasite surface

**Fig 2:**
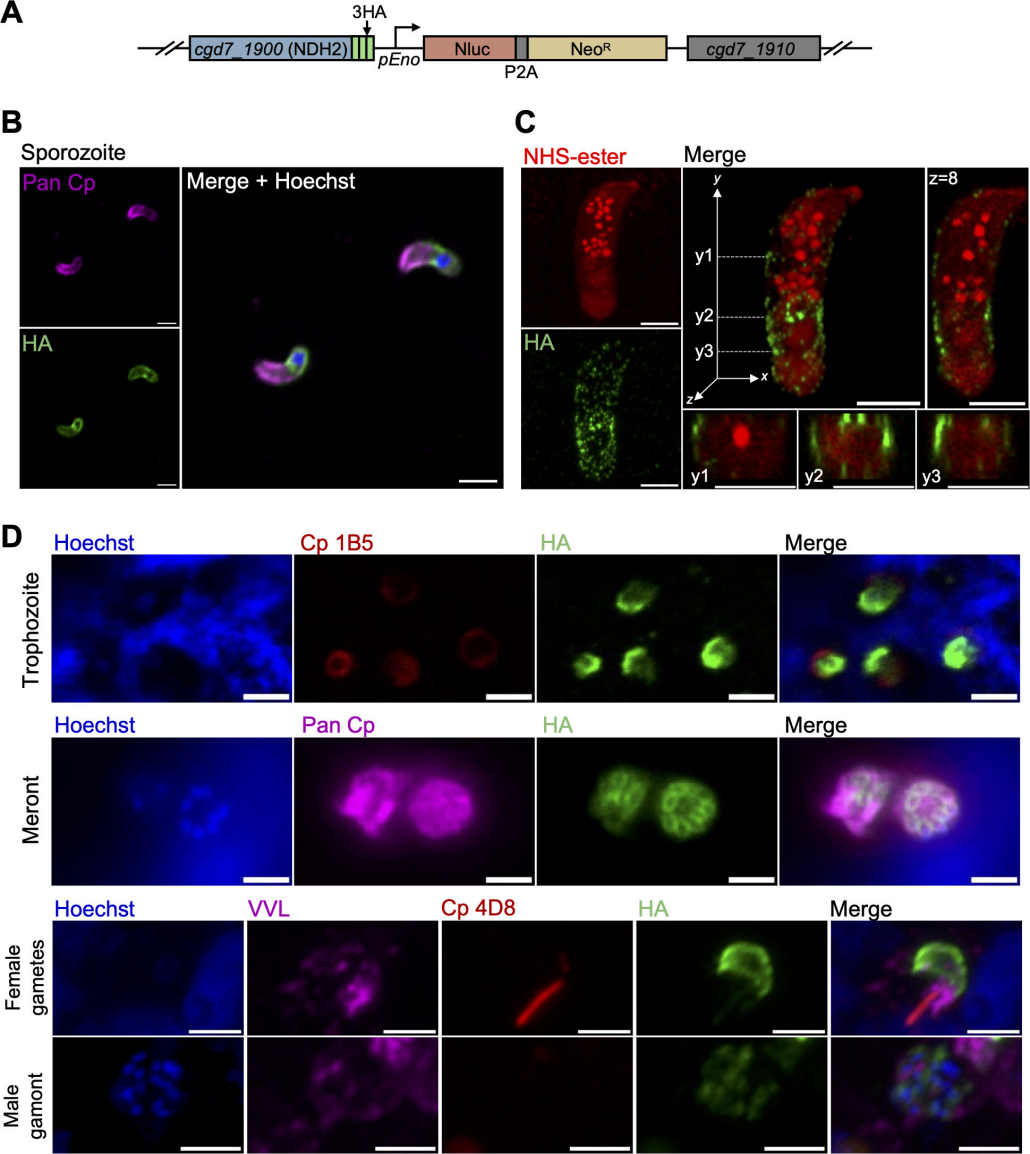
Localization of NDH2 in *C. parvum*. (**A**) Schematic of the NDH2-3HA-tagged endogenous locus in stable transgenic parasites. *C. parvum* sporozoites were co-transfected with NDH2-3HA-Nluc-P2A-Neo^R^ tagging plasmid and CRISPR/Cas9 plasmid containing sgRNA specific to the C-terminal of NDH2. Nluc, Nanoluc luciferase; P2A, split peptide; Neo^R^, neomycin-resistant cassette. (**B**) Immunofluorescence staining of transgenic NDH2-3HA-tagged sporozoites. NDH2-3HA oocysts were excysted using 0.75% sodium taurocholate to release sporozoites. Sporozoites were fixed onto coverslips and stained with rat anti-HA, followed by goat anti-rat IgG Alexa Fluor 488 (green), Pan Cp followed by goat anti-rabbit IgG Alexa Fluor 647 (magenta), and Hoechst (blue) for nuclear staining. Scale bars, 2 µm. (**C**) U-ExM of transgenic NDH2-3HA sporozoites. NDH2-3HA oocysts were excysted using 0.75% sodium taurocholate to release sporozoites. Sporozoites were fixed onto coverslips and expanded in gel for U-ExM. Expanded samples were stained with rabbit anti-HA followed by goat anti-rabbit IgG Alexa Fluor 488 (green), and NHS-ester-594 (red) for parasite staining. Representative z-plane (z = 8) and several y-planes (Y1, Y2, and Y3) are shown in the right and bottom panels, respectively. Scale bars, 5 µm. (**D**) Immunofluorescence staining of transgenic NDH2-3HA-tagged parasites at intracellular stages. HCT-8 cells were infected with NDH2-3HA oocysts. At 24 or 48 hpi, coverslips were fixed and stained with rat anti-HA, followed by goat anti-rat IgG Alexa Fluor 488 (green), mouse mAb Cp 1B5 or Cp 4D8, followed by goat anti-mouse IgG Alexa Fluor 568 (red), Pan Cp followed by goat anti-rabbit IgG Alexa Fluor 647 or VVL, followed by streptavidin Alexa Fluor 647 (magenta) and Hoechst (blue) for nuclear staining. Scale bars, 2 µm.

To investigate the localization of CpNDH2, we employed CRISPR/Cas9 gene editing to add a triple hemagglutinin (3HA) epitope tag to the C terminus (Fig. 2A). The tagging construct also contained a selection cassette consisting of nanoluciferase (Nluc) and neomycin resistance (Neo^R^) jointed by a split peptide motif (P2A) and driven by an enolase promoter (Fig. 2A; Fig. S3A). The CpNDH2-3HA tagging plasmid was co-transfected into excysted sporozoites with a CRISPR/Cas9 plasmid containing a gene-specific sgRNA targeting Cp*NDH2*. Transfected parasites were selected by paromomycin in Ifng^-/-^ (GKO) mice, followed by the second round of selection and amplification in Nod scid gamma (NSG) mice. The genotype of transgenic parasites was validated using diagnostic PCR to detect insertion of the tag at the endogenous locus using DNA extracted from mice fecal pellets at 30 days post-infection (dpi) (Fig. S3B). Growth of the CpNDH2-3HA tagging strain was assessed by testing the luminescence signal from the Nluc gene from NSG fecal pellets from 3 to 30 dpi (Fig. S3C). To visualize the location of CpNDH2, we performed immunofluorescence assays (IFA) using the CpNDH2-3HA tagged parasite. CpNDH2 protein expression was detected in all life cycle stages, where it showed a surface membrane staining pattern (Fig. 2B and D). Parasites were co-stained with a polyclonal rabbit antibody to *C. parvum* called Pan Cp, the lectin VVL, or several mouse mAbs that we have previously characterized based on their reaction to different life cycle stages of *C. parvum* grown *in vitro* (31). Staining with Pan Cp revealed a surface pattern in sporozoites that overlaps with the pattern of CpNDH2 (Fig. 2B). Due to the diminutive dimensions of the parasite, we utilized ultrastructure expansion microscopy (U-ExM), which can increase the specimen size by up to fourfold (32), to test whether the cell membrane is the exclusive localization site of CpNDH2. After expansion, CpNDH2 exhibited a scattered, punctate staining pattern, which may result from stretching of the parasite membrane during the expansion process (Fig. 2C). Membrane-associated localization was consistently observed across the z-plane and multiple y-planes throughout the entire sporozoites (Fig. 2C). We also colocalized CpNDH2 with a marker for the periphery of the feeder organelle that stains a donut-shaped ring at the host-parasite interface, while NDH2 occupies the much larger surface of the trophozoite (Fig. 2D). In mature meronts, the surface staining of CpNDH2 highlighted the outline of individual merozoites in a cluster that was co-stained with Pan Cp (Fig. 2D). Interestingly, NDH2 showed an asymmetric surface staining pattern on macrogamonts recognized by the fibrillar staining with mAb 4D8, while it was highly diffuse in microgametocytes, detected by the many small nuclei stained with Hoechst (Fig. 2D). Due to the atypical location of NDH2 in *C. parvum*, we repeated the staining using the same anti-HA antibody and parasite markers in wild-type *C. parvum*, which ruled out that the observed staining was due to nonspecific binding of the antibodies (Fig. S4).

### CpAOX exhibits a mitosome-like localization pattern

**Fig 3:**
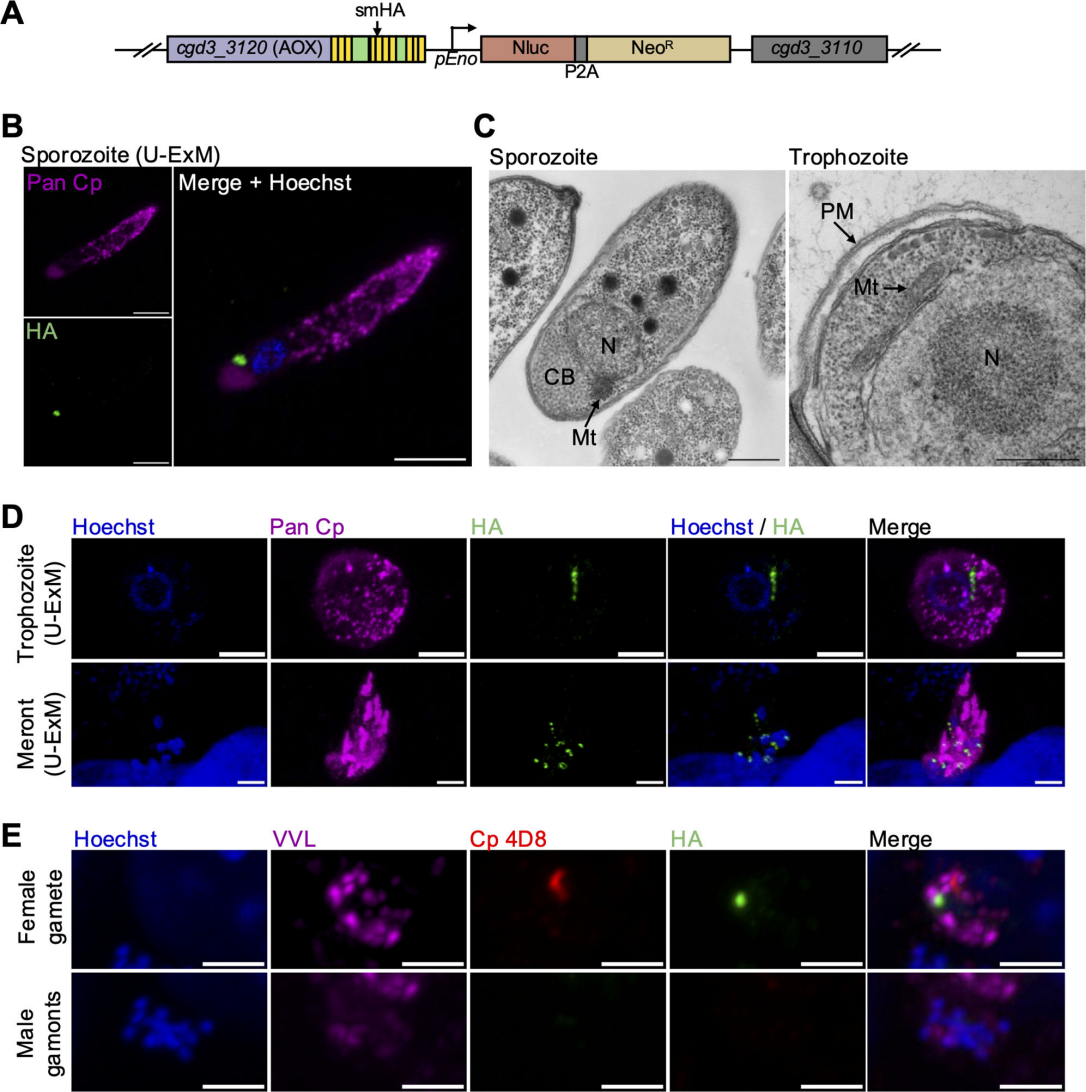
Localization of AOX in *C. parvum*. (**A**) Schematic of the AOX-smHA-tagged endogenous locus in stable transgenic parasites. *C. parvum* sporozoites were cotransfected with AOX-smHA-Nluc-P2A-Neo^R^ tagging plasmid and CRISPR/Cas9 plasmid containing sgRNA specific to the C-terminal of AOX. smHA, spaghetti-monster HA. (**B**) U-ExM of transgenic AOX-smHA sporozoites. AOX-smHA oocysts were excysted using 0.75% sodium taurocholate to release sporozoites. Sporozoites were fixed onto coverslips and expanded in gel for U-ExM. Expanded samples were stained with rabbit anti-HA, followed by goat anti-rabbit IgG Alexa Fluor 488 (green), rabbit Pan Cp, followed by goat anti-rabbit IgG Alexa Fluor 647 (magenta), and Hoechst (blue) for nuclear staining. Scale bars, 5 µm. (**C**) Transmission electron micrographs of the mitosome in the *C. parvum* sporozoite and trophozoite. Mt, mitosome; N, nucleus; CB, crystaloid body; PVM, parasitophorous vacuole membrane. Scale bars, 500 nm. (**D**) U-ExM of transgenic AOX-smHA parasites at intracellular stages. HCT-8 cells were infected with AOX-smHA oocysts. At 24 hpi, infected cells were fixed and expanded in gel for U-ExM. Expanded samples were stained with rabbit anti-HA, followed by goat anti-rabbit IgG Alexa Fluor 488 (green), rabbit Pan Cp, followed by goat anti-rabbit IgG Alexa Fluor 647 (magenta), and Hoechst (blue) for nuclear staining. Scale bars, 5 µm. (**E**) Immunofluorescence staining of transgenic NDH2-3HA-tagged parasites at sexual stages. HCT-8 cells were infected with NDH2-3HA oocysts. At 48 hpi, coverslips were fixed and stained with rat anti-HA followed by goat anti-rat IgG Alexa Fluor 488 (green), mouse mAb Cp 4D8 followed by goat anti-mouse IgG Alexa Fluor 568 (red), VVL followed by streptavidin Alexa Fluor 647 (magenta), and Hoechst (blue) for nuclear staining. Scale bars, 2 µm.

We also generated an epitope-tagged strain to localize CpAOX. Due to its relatively low expression, we tagged CpAOX with a spaghetti monster HA tag (smHA) (33), which contains 10 separate HA epitopes on a non-fluorescent GFP protein backbone (Fig. 3A; Fig. S3D). The genotype CpAOX-smHA tagging strain was validated using diagnostic PCR with primers specific to the modified endogenous locus (Fig. S3E). Amplification of the CpAOX-smHA strain in NSG mice exhibited comparable nanoluciferase levels to those of CpNDH2-3HA parasites, although the differences in epitopes limit direct comparisons of growth efficiency (Fig. S3C and F). To visualize the location of CpAOX, we performed IFA on normal samples (Fig. S5A) and those treated for expansion microscopy due to the small size of the mitosome (Fig. 3B and D). Laser scanning confocal microscopy of expansion samples revealed that CpAOX was localized in a small punctate dot posterior to the nucleus in sporozoites (Fig. 3B). CpAOX also appeared as a punctate dot in trophozoites seen in normal samples (Fig. S5A, upper panel), but had an elongated oval structure in trophozoites observed in expansion samples (Fig. 3D, upper panel). These locations are highly consistent with the position of the *C. parvum* mitosome in these two stages as observed by electron microscopy (Fig. 3C, see enlarged images in Fig. S6). Additionally, CpAOX appeared as punctate dots associated with the nuclei in mature meronts (Fig. 3D, lower panel, Fig. S5A, lower panel). We attempted immuno-EM using the CpAOX-smHA tagged strain, but the results were inconclusive, likely due to the epitope not surviving fixation. As an alternative, we performed co-staining with MitoTracker, which accumulates in the mitochondrion based on the membrane potential. Co-staining with MitoTracker showed a partial overlap between CpAOX and MitoTracker in mature meronts, where the mitosome is a small focal point near the nucleus (Fig. S5B). Interestingly, CpAOX was detected as a single punctate dot in macrogamonts, recognized by mAb Cp 4D8, but was absent in microgametes, detected by many small nuclei stained with Hoechst (Fig. 3E).

### Neither AOX nor NDH2 is essential for parasite growth

**Fig 4:**
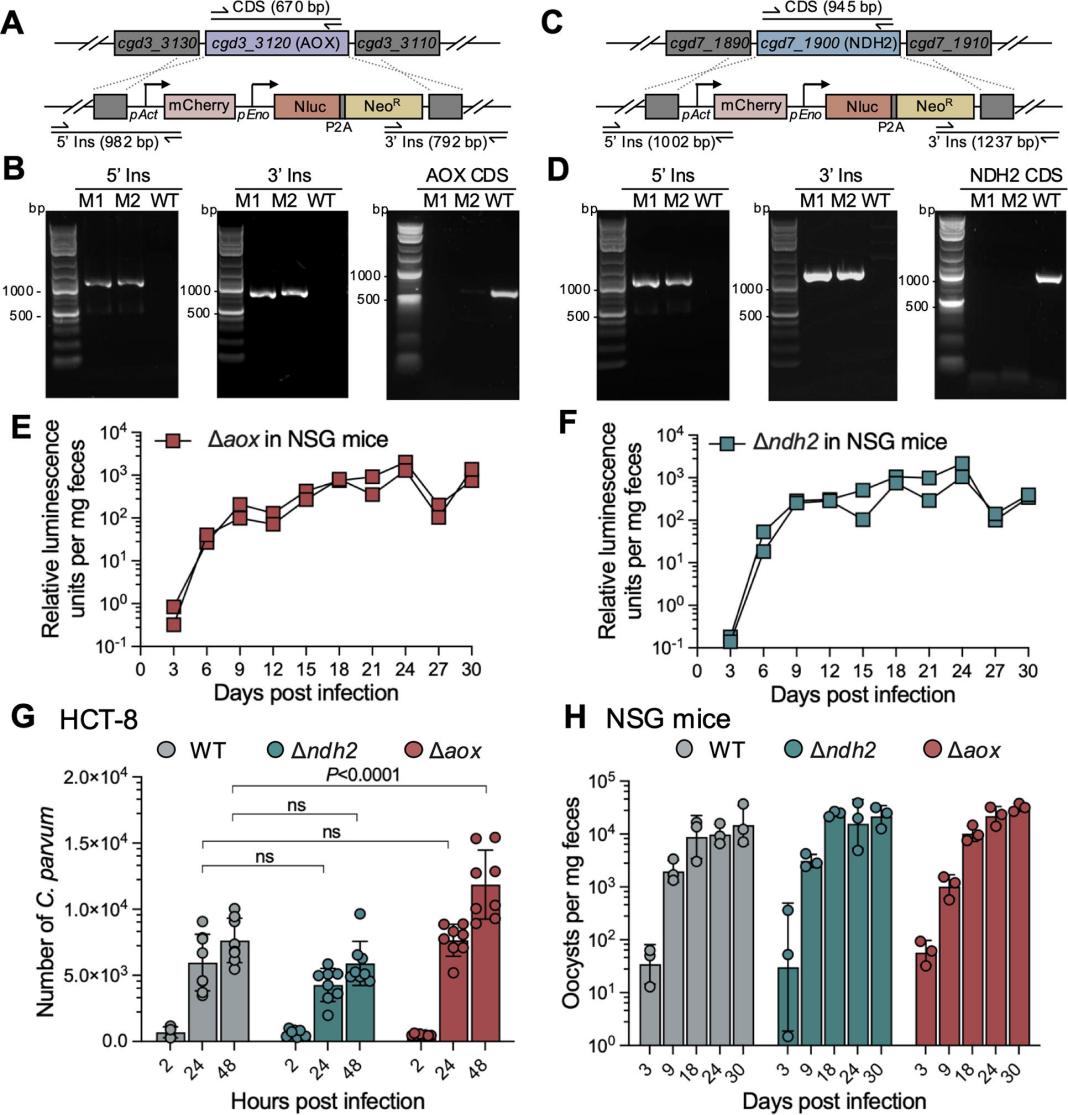
Testing the essentiality of NDH2 and AOX for parasite growth. (**A**) Diagram of the strategy to construct Δ*aox* transgenic parasites. Construct was designed to replace the AOX locus with an mCherry and Nluc-P2A-Neo^R^ cassette. The top line shows the genomic locus, and the bottom line the successfully targeted transgenic locus. sgRNA, small guide RNA. 5′ Ins and 3′ Ins refer to diagnostic PCR fragments. (**B**) Genotype analysis of the Δ*aox C. parvum* strain by PCR. M1 and M2, Δ*aox* parasites from two NSG mice. The products 5′ Ins and 3′ Ins are specific for the 5′ CRISPR integration and the 3′ CRISPR integration at the genomic locus of AOX, respectively. The product CDS is specific for the coding sequence of AOX. Primers are defined in Table S2. (**C**) Diagram of the strategy to construct Δ*ndh2* transgenic parasites. (**D**) Genotype analysis of the Δ*ndh2 C. parvum* strain by PCR. M1 and M2, Δ*ndh2* parasite from two NSG mice. The products 5′ Ins and 3′ Ins are specific for the 5′ CRISPR integration and the 3′ CRISPR integration at the genomic locus of NDH2, respectively. The product CDS is specific for the coding sequence of NDH2. Primers are defined in Table S2. (**E**) Relative luminescence per milligram of feces from NSG mice challenged by Δ*aox* parasites. Each data point represents a single fecal pellet, and each connecting line represents an individual infected NSG mouse. (**F**) Relative luminescence per milligram of feces from NSG mice challenged by Δ*ndh2* parasites. Each data point represents a single fecal pellet, and each connecting line represents an individual infected NSG mouse. (**G**) *In vitro* growth assay of WT, Δ*aox,* and Δ*ndh2* strains. Relative fluorescence of *C. parvum* was quantified using a cell imaging reader. Values are plotted as the means ± SD. Statistical analysis was performed using two-way ANOVA with Tukey’s multi-comparison test of data from two independent experiments. ns, not significant. (**H**) *In vivo* growth assay of WT, Δ*aox,* and Δ*ndh2* strains. The NSG mouse was challenged with 2 × 10^4^ oocysts via oral gavage. Three NSG mice were infected with each *C. parvum* strain. Fecal samples were collected at D3, D9, D18, D24, and D30 pi. DNA was extracted from fecal samples, and oocyst shedding from mice was evaluated using qPCR with primers specific to *C. parvum* glyceraldehyde-3-phosphate dehydrogenase. Values are plotted as the means ± SD. Statistical analysis was performed using two-way ANOVA with Tukey’s multi-comparison test of data (*n* = 3). No statistically significant difference was detected between wild-type and KO strains at respective time points.

To test the essentiality of AOX or NDH2 for *C. parvum* growth, we generated knockout (KO) strains to deplete either AOX (Fig. 4A) or NDH2 (Fig. 4C) from parasites using CRISPR/Cas9. The targeted gene was replaced by a mCherry expression cassette driven by the *C. parvum* actin promoter and the Nluc-P2A-Neo^R^ selection marker described above. Following selection and amplification in GKO and NSG mice, we successfully obtained both Δ*aox* and Δ*ndh2* strains. Deletion of the targeted genes and insertion of selective marker in specific genomic sites were validated using diagnostic PCR with genomic DNA from fecal samples from NSG mice at 30 dpi (Fig. 4B and D). These PCR results confirmed the complete deletion of either AOX or NDH2 from the parasite genome in the KO strains. To further confirm the loss of AOX or NDH2 at the mRNA level in the respective KO strains following mouse passage, HCT-8 cells were infected with either wild-type or KO parasites, and total RNA was extracted for analysis. Both RT-qPCR and RT-PCR confirmed the absence of AOX or NDH2 transcripts in the KO parasites (Fig. S7). The fitness of KO strains was similar to that of tagging strains based on nanoluciferase expression, suggesting there is little or no deficiency in growth due to loss of either gene (Fig. 4E and F; Fig. S3C and F).

To further characterize the growth of KO strains, we performed growth assays *in vivo* in NSG mice and *in vitro* with HCT-8 cells to compare the growth fitness of KO strains to wild-type parasites. To reduce the effect of host adaptation, we passaged the calf-derived wild-type parasites in NSG mice and collected oocysts shed from mice. *In vitro* parasite growth in HCT-8 cells was determined via immunofluorescent staining followed by quantification using plate-based imaging. The result of the *in vitro* growth assay suggested a similar growth of the Δ*ndh2* strain to the wild-type strain, while the Δ*aox* strain exhibited a moderate growth enhancement at 48 h post-infection (hpi) (Fig. 4G). However, no difference in oocyst shedding was observed when comparing the mice challenged by either the KO strain with mice infected by wild-type parasites from 3 to 30 dpi (Fig. 4H).

### AOX is not required for MitoTracker staining or for growth inhibition by SHAM and 8-HQ

**Fig 5:**
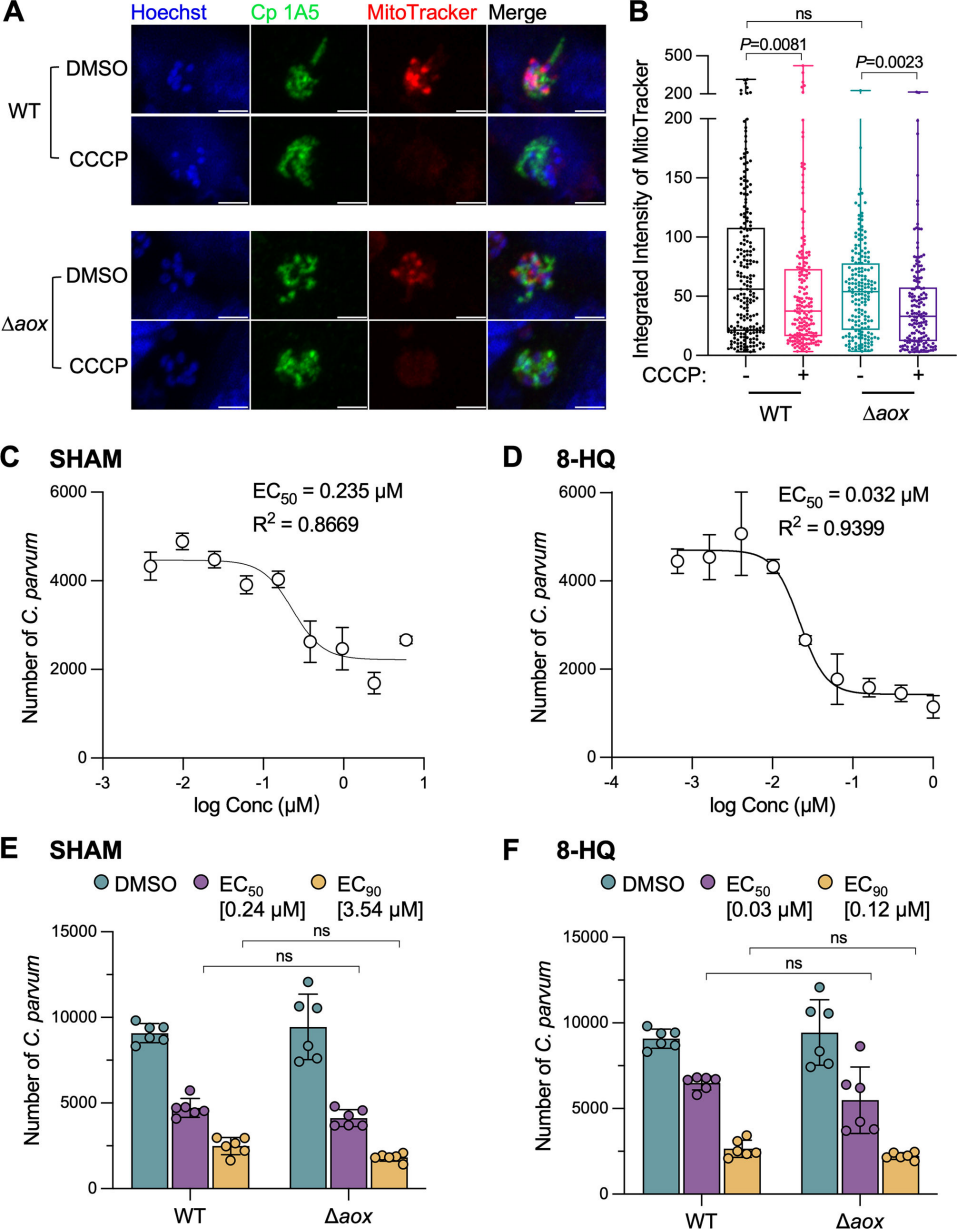
Mitosome membrane potential and inhibitor sensitivity of ∆*aox* strain parasites. (**A**) Fluorescence microscopy images of *C. parvum* in HCT-8 cultures treated with 1% dimethyl sulfoxide (DMSO) or 10 µM CCCP for 2 h (from 22 to 24 hpi). Parasites were labeled with apical marker mAb Cp 1A5 (green), MitoTracker Red CMXRos (red), and nuclei were stained with Hoechst (blue). Scale bar, 2 µm. (**B**) Distribution of MitoTracker intensity for ∆*aox* parasites. *C. parvum* in HCT-8 cultures was treated with 1% DMSO or 10 µM CCCP for 2 h (from 22 to 24 hpi). Mature meronts were defined by staining with mAb Cp 1A5 that localizes the apex in mature merozoites (31, 35). Integrated intensity of MitoTracker staining was collected from at least 150 parasites from two independent experiments. Statistical analysis was performed using the Kruskal-Wallis test. ns, not significant. (**C**) Dose-response of *C. parvum* growth vs. concentrations of SHAM. Drugs were tested in a 9-point 1:2.5 serial dilution series starting at 6 µM. EC_50_ and R squared values were calculated in GraphPad Prism 9 using a nonlinear regression curve fit. (**D**) Dose-response of *C. parvum* growth vs. concentrations of 8-HQ. Drugs were tested in a 9-point 1:2.5 serial dilution series starting at 1 µM. (**E**) Relative growth of WT and AOX-KO parasites treated with SHAM at EC_50_ or EC_90_. Plate-based growth assay using HCT-8-infected cells that were fixed and stained with rat anti-HA, followed by goat anti-rat IgG Alexa Fluor 488, and imaging using a Cytation 3.0 plate imager. Values are plotted as the means ± SD. Statistical analysis was performed using two-way ANOVA with Tukey’s multi-comparison test of data from two independent experiments. ns, not significant. (**F**) Relative growth of WT and AOX-KO parasites treated with 8-HQ at EC_50_ or EC_90_. Plate-based growth assay using HCT-8-infected cells that were fixed and stained with rat anti-HA, followed by goat anti-rat IgG Alexa Fluor 488 and imaging using a Cytation 3.0 plate imager. Values are plotted as the means ± SD. Statistical analysis was performed using two-way ANOVA with Tukey’s multi-comparison test of data from two independent experiments. ns, not significant.

*C. parvum* meronts stained with MitoTracker exhibit a pattern consistent with the mitosome, and this signal is disrupted by carbonyl cyanide 3-chlorophenylhydrazone (CCCP), consistent with the staining being due to a membrane potential (34). To clarify the role of AOX in this staining pattern, we compared MitoTracker Red CMXRos staining between ∆*aox* and wild-type strains. For these studies, we used mature meronts defined by staining with mAb 1A5 (31, 35) and where the individual mitosomes within separate merozoites are discretely stained by MitoTracker Red CMXRos. Both qualitative images obtained by microscopy and quantitative analysis of the MitoTracker signal intensity indicated that no significant difference in staining between the ∆*aox* and wild-type parasites (Fig. 5A and B). Additionally, the intensity of MitoTracker Red CMXRos was significantly decreased by treatment with CCCP for both wild type and the ∆*aox* mutant (Fig. 5A and B). The finding that loss of AOX has no effect on MitoTracker staining suggests that it is not required for the establishment of the membrane potential.

Previous studies have reported that two AOX inhibitors, SHAM and 8-HQ, inhibit the growth of *C. parvum* (23). This sensitivity, combined with the unique presence of this enzyme in the parasite and absence in the host, was used as a rationale to suggest that AOX might be a good drug target (23). To further investigate the sensitivity of SHAM- and 8-HQ-mediated inhibition, we conducted dose-response assays using drugs that were diluted in a 9-point 1:2.5 series, starting at 6 µM for SHAM and 1 µM for 8-HQ, and used to treat HCT-8 cells challenged by wild-type *C. parvum*. We determined EC_50_ values of SHAM and 8-HQ for *C. parvum* growth inhibition of 0.235 µM and 0.032 µM, respectively (Fig. 5C and D). The EC_90_ values of each drug were calculated via a computational tool (https://www.graphpad.com/quickcalcs/Ecanything1/), providing estimates of 3.54 µM for SHAM and 0.12 µM for 8-HQ. To compare the sensitivity of ∆*aox* and wild-type strains, we treated parasites grown in HCT-8 cells with SHAM or 8-HQ at EC_50_ or EC_90_ for 24 h. The growth assay results indicated that neither of the drugs exhibited different effectivity to ∆*aox* compared with that of wild-type parasites (Fig. 5E and F).

## DISCUSSION

*C. parvum* and *C. hominis*, the most common species infecting humans, possess a relict mitochondria-related organelle, called the mitosome, which is highly reduced in size, morphology, and functionality. Comparative genomic analysis of *C. parvum* indicates that mitochondrial metabolism-related proteins are restricted to Fe-S biosynthesis, ubiquinone biosynthesis, and an alternative ETC, including MQO, NDH2, and AOX. Due to their absence in mammalian hosts, NDH2 and AOX have been proposed to be potential drug targets for cryptosporidiosis. In this study, we focused on the characterization of NDH2 and AOX and clarified that only AOX exhibited a mitosome-like localization, whereas NDH2 was found at the surface membrane or IMC. Moreover, deletion of NDH2 or AOX showed a minor effect on growth *in vitro* and no impact on the growth fitness of *C. parvum* in mice. Furthermore, although AOX inhibitors SHAM and 8-HQ have been reported to suppress the growth of *C. parvum in vitro*, our finding that ∆*aox* parasites are similarly sensitive to these inhibitors rules out AOX as the target of these compounds. Collectively, these findings force a revision to the proposed model for how the mitosome membrane potential is generated and also deprioritize NDH2 and AOX as potential drug targets.

Type II NDH enzymes sit in the inner or outer leaflet of the inner mitochondrial membrane and have been reported in mitochondria of plants, fungi, as well as protists, but not in mammals (12, 36). Given their essentiality in respiration and metabolism in bacterial pathogens and absence in mammalian hosts, these enzymes have been proposed as potential novel therapeutic targets (37–40). NDH2 proteins are also predicted to be encoded in the genome of apicomplexans, which lack a canonical complex I (4, 7, 12). Two isoforms of NDH2 are found in *T. gondii*, both of which are internal, monomeric proteins facing their active sites to the mitochondria matrix (13). Functional analysis suggested that these two isoforms are individually non-essential; however, deletion of either isoform decreased the growth rate and reduced the mitochondrial membrane potential in *T. gondii* (13). *Plasmodium* spp. express a single NDH2 protein, which was initially reported to be sensitive to diphenylene iodonium chloride (DIC) that depolarizes the mitochondrial membrane potential, leading to parasite death (17). However, this finding was challenged by a later study using recombinantly expressed PfNDH2, which found that it is not sensitive to DIC (41). Moreover, a recent study depleted PfNDH2 using CRISPR/Cas9-based genome editing and demonstrated that this protein is dispensable in *P. falciparum* and that the mutant is still sensitive to inhibitors of the ETC, suggesting that they target other components of the respiratory complexes (42).

In the present study, we were surprised to discover that NDH2 in *C. parvum* is predominantly expressed at the parasite membrane. Due to limitations in the resolution of light microscopy, we are unable to differentiate between a surface membrane localization and localization in the IMC, as reported by the HyperLOPIT study (29). Given the putative enzymatic activity of NDH2, the conversion of NADH to NAD^+^ and H^+^, it is unclear why this activity would be required at the parasite surface and what reductive electron acceptor would be involved at this interface. Regardless of its exact function, loss of this protein did not significantly affect parasite growth, either *in vitro* or *in vivo*, indicating that NDH2 is dispensable for *C. parvum* growth. NDH2 is also non-essential for *P. falciparum* growth in red blood cells (42), although it likely plays a different role in this parasite, which contains a very different ETC.

Currently, AOX has only been identified in non-mammal organisms, nominating it a potential drug target. Most plants and fungi contain both a canonical respiration pathway using complex III and IV and an alternative respiration pathway involving AOX. In some fungi and plants, AOX genes are constitutively transcribed at a low basal level without detectable protein and enzyme activity, whereas its expression can be activated upon the inhibition of the canonical respiration pathway or the presence of oxidative stress in these organisms (43, 44). Unlike NDH2, an AOX-like protein was not identified in the genomes of either *T. gondii* or *P. falciparum*. The most extensively studied AOX expressed in a protozoan parasite is the *Trypanosoma brucei* AOX (TAO), which demonstrates a developmentally regulated expression in the *T. brucei* life cycle (22). As the only terminal oxidase of the mitochondrial ETC in the bloodstream *T. brucei*, AOX exhibits a significantly higher mRNA level and stability as well as protein abundance, compared to the procyclic form (45). Reduction in TAO mRNA level using RNAi or treatment with AOX inhibitor SHAM inhibits *T. brucei* growth (46). Similarly*,* AOX is also the only identified terminal oxidase in the parasite genome in *C. parvum,* and previous studies have suggested the sensitivity of *C. parvum* to AOX inhibitors makes this a potential target for the development of therapeutics (23).

In the present study, we used U-ExM to examine the localization of AOX in *C. parvum* and observed staining patterns that are consistent with the mitosome, which is an elongate oval in trophozoites and a single small spherical body in mature merozoites. The distribution of AOX revealed by IFA closely matches the localization of mitosome observed by TEM—juxtanuclear, posterior dot in sporozoites and mature merozoites, and elongated oval shape in trophozoites. Recent studies using immuno-EM localization also verified that AOX is localized to the mitosome (47). This localization is also consistent with HyperLOPIT data, indicating that AOX cofractionates with other mitosome proteins (29). However, CpAOX was only partially co-localized with MitoTracker, and this incomplete pattern might result from several mechanisms. First, it may reflect the compartmentalization of CpAOX in one segment of the mitosome. A similar distribution pattern has been reported for the mitochondrial protein adenine nucleotide carrier in *Toxoplasma* (48). Consistent with this possibility, three-dimensional reconstructions of the mitosome reveal that it is not a simple sphere but rather it contains internal membrane folds, suggesting compartmentalization (49). Alternatively, the membrane potential of the *C. parvum* mitosome is likely lower than that of conventional mitochondria, due to the absence of key respiratory complexes for proton pumping. Under such circumstances, MitoTracker may also accumulate in the ER, which retains a weak membrane potential. Consistent with this hypothesis, prior studies using MitoTracker to stain *C. parvum* have reported broad, diffuse labeling that is more reminiscent of the ER than mitosome (23, 50). Under the condition utilized here, we observe much more discrete staining with MitoTracker, and yet only a portion of this signal overlapped with AOX. Future studies will be necessary to clarify these alternative explanations and to improve approaches for measuring the membrane potential in the mitosome.

Surprisingly, deletion of AOX using CRISPR/Cas9-based genome editing did not show any impact on the asexual development, although a modest growth enhancement was observed in the AOX-depleted parasite during the late stages of the life cycle. However, no significant differences were observed in oocyst shedding from mice infected with ∆*aox C. parvum* compared to wild-type parasites, indicating that AOX is non-essential for parasite growth. MitoTracker staining demonstrated no detectable difference between ∆*aox C. parvum* and wild-type parasites, suggesting that AOX may not be required for establishment of the mitosome membrane potential. One limitation of these studies is that co-staining of AOX and MitoTracker shows only partial overlap, which may reflect the compartmentalized AOX localization or MitoTracker accumulation in another organelle, such as the ER. Nonetheless, the absence of a growth defect in the ∆*aox* strain indirectly suggests that AOX is not essential for maintaining the mitosome membrane potential, as this gradient is typically required for protein import across the inner membrane of mitochondria (1). Previous studies have shown that AOX inhibitors, SHAM and 8-HQ, inhibit the *C. parvum* growth *in vitro* (23, 24), which was confirmed by the dose-response assays in this study. However, ∆*aox* parasites showed sensitivities to SHAM and 8-HQ that were similar to wild-type parasites, indicating that AOX is not the primary target of SHAM and 8-HQ in *C. parvum*. Previous studies have also shown that these inhibitors act on *Toxoplasma gondii*, even though it does not encode AOX (23), further supporting the conclusion that they act on alternative targets.

Our study revealed that NDH2 is surface membrane localized and non-essential in *C. parvum*. Although AOX exhibits a mitosome localization pattern, it is also dispensable for parasite growth and mitosome membrane potential generation. The AOX-KO mutant and wild-type parasites were equally sensitive to the AOX inhibitor SHAM and 8-HQ. These findings challenge the previous proposal that AOX and NDH2 are potential drug targets of future therapeutic development for cryptosporidiosis. Additionally, the finding that the proposed alternative ETC is nonessential indicates that the membrane potential in the mitosome must be generated by an alternative means. The predicted localization of the MQO proteins in *C. parvum* in the nucleus or cytoplasmic fraction, combined with the non-essentiality of AOX, reduces their potential importance in contributing to the membrane potential. Previous studies have suggested that TH, which can mediate proton pumping, may contribute to the mitosome membrane potential (28). However, the *C. parvum* TH protein encoded by *cgd8_2330* is predicted to localize in micronemes (29). It remains possible that the remaining TH protein encoded by *cgd1_990* is mitosome localized, although predictions from MitoProt do not support this localization (28). Alternatively, *C. parvum* contains an ADP/ATP carrier protein that is normally in the mitochondria and predicted to be in the mitosome (51). The absence of oxidative phosphorylation in *C. parvum* suggests that the ADP/ATP carrier protein may work in reverse to transport ATP into the mitosome, thus providing a source of energy for critical reactions such as iron-sulfur cluster biosynthesis (7, 10). Previously, it was proposed that the import of ATP^−4^ in exchange for ADP^−3^ creates a charge asymmetry that generates a membrane potential in the *C. parvum* mitosome (52), similar to petite mutants in human cells lacking a functional ETC (53). Further studies are needed to resolve the localization and function of the ADP/ATP carrier protein in *C. parvum* and to resolve the mechanism by which the membrane potential is generated.

## MATERIALS AND METHODS

### Animal studies

Animal studies using mice were approved by the Institutional Animal Studies Committee (School of Medicine, Washington University in St. Louis). *Ifng*^-/-^ mice (referred to as GKO) (002287; Jackson Laboratories), and Nod scid gamma mice (referred to as NSG) (005557; Jackson Laboratories) were bred in-house at Washington University School of Medicine and were separated by sex after weaning. Mice were reared in a specific-pathogen-free facility on a 12 h:12 h light-dark cycle and water *ad libitum*. For selection and amplification of transgenic *C. parvum* parasites, 8- to 12-week-old male or female mice were used, and water was replaced with filtered tap water containing 16 g/liter paromomycin sulfate salt (Goldbio, #P-700-100). During infection, animals with more than 20% body weight loss or appearing debilitated were humanely euthanized.

For monitoring parasite growth *in vivo*, NSG mice were challenged with 2 × 10^4^ parasites by oral gavage, and animals were maintained on normal feed and water. Mouse fecal pellets were collected every 3 days post-infection. Mice were euthanized when they lost more than 20% body weight during infection.

### HCT-8 cell culture

Human ileocecal adenocarcinoma cells (HCT-8 cells; ATCC CCL-244) were cultured in RPMI 1640 medium (ATCC modification; Gibco) supplemented with 10% fetal bovine serum. The HCT-8 cells were determined to be mycoplasma negative using the e-Myco plus kit (Intron Biotechnology).

### Parasite preparation

The *C. parvum* isolate (AUCP-1) was maintained by repeated passage in male Holstein calves and purified from fecal material, as described previously (54). Purified oocysts were stored at 4°C in 50 mM Tris-10 mM EDTA (pH 7.2) for up to 6 months before use. Oocysts were prepared with 40% bleach before infection, as described previously (55). Briefly, purified oocysts were incubated with 40% bleach in Dulbecco’s phosphate-buffered saline (DPBS; Corning Cellgro) for 10 min on ice. Oocysts were then washed four times in DPBS containing 1% (wt/vol) bovine serum albumin (BSA; Sigma) and resuspended in 1% BSA/DPBS. For some experiments, oocysts were excysted prior to infection by incubating the oocysts with 0.75% (wt/vol) sodium taurocholate (Sigma) at 37°C for 60 min.

The transgenic parasite was purified from NSG mice feces using saturated sodium chloride (NaCl) flotation as described in reference (56). Briefly, fecal pellets from infected mice were mixed and washed in cold distilled water followed by centrifugation at 2,000 × *g* for 10 min at 4°C. The pellet was resuspended in cold distilled water and mixed with flotation medium (saturated NaCl solution, d = 1.18 g/mL, supplemented with 0.2% Tween-20). Cold distilled water was overlaid to prevent destruction of oocysts resulting from extended exposition to the hypertonic NaCl solution. The tube was centrifuged at 2,000 × *g* for 30 min at 4°C. Oocysts accumulated in a white thin layer at the bottom of the distilled water phase. After collection, oocysts were washed three times with 1× PBS followed by centrifugation at 2,000 × *g* for 10 min at 4°C. The resulting pellet contained the accumulated oocysts which were resuspended in 1× PBS and quantified using C-Chip hemocytometer (INCYTO).

### CRISPR/Cas9-based genome editing

To generate tagging plasmids, a 5′ homology region from the C terminus of the protein (397 bp) containing a protospacer adjacent motif (PAM) and a 3′ homology region from the 3′ UTR of the gene (400 bp) were amplified from *C. parvum* genome DNA by PCR. The triple hemagglutinin (3 HA) and spaghetti monster HA (smHA) epitope tags were amplified from pCpGT1-3HA and pCpGT2-smHA, respectively (32). The previously described Nluc-P2A-neoR reporter and the pUC19 backbone were amplified from pCpGT1-3HA plasmid (32). The tagging plasmids were generated by Gibson assembly (New England Biolabs, #E2611L) of components described above. PAM sites were mutated by PCR amplification using primers to edit the sequence followed by treatment with KLD enzyme kit (New England Biolabs, #M0554S).

To generate repairing templates for gene deletions, homology repair fragments flanking the mCherry-Nluc-P2A-Neo^R^ cassette with 50 bp 5′UTR and 3′’UTR homology regions for the genes of interest were PCR amplified from pINS1-mCherry-Nluc-P2A-neo-INS1 (55) with primers containing appropriate gene-specific homology regions. These plasmids express a neomycin resistance gene, called neo, which is a bacterial gene that encodes an enzyme called neomycin phosphotransferase II (NPT II). This gene confers resistance to aminoglycoside antibiotics including paromomycin used in this study.

To generate the CRISPR/Cas9 plasmid, a single-guide RNA (sgRNA) targeting 3′ end of target genes was designed using the eukaryotic pathogen CRISPR guide RNA/DNA design tool (http://grna.ctegd.uga.edu/). The pCRISPR/Cas9 backbone was amplified from previously described pACT1:Cas9-GFP, U6:sgINS1 (55). pCRISPR/Cas9-sgRNA plasmids were generated with the designed sgRNA and pCRISPR/Cas9 backbone, using Q5 site-directed mutagenesis (New England Biolabs, #E0554). The same pCRISPR/Cas9-sgRNA plasmid was used for tagging and deletion of the gene of interest.

All the primers used for fragment amplifications are listed in Table S2. All the plasmids generated in this study are described in Table S3.

For transfection, oocysts (1.25 × 10^7^ per transfection) were excysted as described above, and sporozoites were collected by centrifugation at 2,500 rpm for 3 min and resuspended in SF buffer (Lonza) containing 50 mg of tagging plasmid or 30 mg of linear targeting template and 30 mg CRISPR/Cas9 plasmid in a total volume of 100 mL. The mixtures were then transferred to a 100 mL cuvette (Lonza) and electroporated on an AMAXA 4D-Nucleofector system (Lonza) using program EH100. Electroporated sporozoites were transferred to cold DPBS and kept on ice before infecting mice. All the repairing templates and CRISPR/Cas9 plasmids used for transgenic strains are specified in Table S4.

### Selection and amplification of transgenic parasites in mice

GKO mice were used for the first round of transgenic parasite selection. Each mouse was orally gavaged with 200 µL of 8% (wt/vol) sodium bicarbonate 5 min prior to infection. Mice were then gavaged with 2.5 × 10^7^ electroporated sporozoites. All mice received drinking water with 16 g/L paromomycin continuously from 1 dpi, based on previously published protocol (57). Fecal pellets were collected to begin at 9–15 dpi, after which animals were euthanized by CO_2_ asphyxiation according to the animal protocol guidelines. Fecal pellets were stored at −80°C for qPCR or at 4°C for luciferase assays or for isolating oocysts for subsequent infections. A second round of amplification was performed by orally gavaging NSG mice using a fecal slurry from GKO mice described above. The fecal pellets were transferred to a 1.7 mL microcentrifuge tube, ground with a pestle, diluted by the addition of 1 mL cold 1× DPBS, vortexed for 30 s followed by a centrifugation at 200 rpm for 10 min to pellet large particulates. Oocysts in the supernatant were counted using a C-Chip hemocytometer and diluted in 1× DPBS. 2 × 10^4^ oocysts were gavaged into one NSG mouse. Infected NSG mice were treated with 16 g/L paromomycin drinking water for the entirety of the experiment. Fecal pellets for qPCR and luciferase assay were collected every 3 days starting 3 dpi, and fecal pellets for purification were collected every day starting at 12 dpi and stored at 4°C. Oocyst purification from NSG feces was as described above. Purified oocysts were stored in PBS at 4°C and used within 6 months of extraction.

### Luciferase assay

Luciferase assays were performed using the Nano-Glo Luciferase assay kit (Promega). Mouse fecal pellets were collected and weighed in 1.7 mL microcentrifuge tubes, ground with a pestle. Glass beads (3 mm; Fisher Scientific) and 1 mL fecal lysis buffer (50 mM Tris pH 7.6, 2 mM DTT, 2 mM EDTA pH 8.0, 10% glycerol, and 1% Triton X-100 prepared in water) (58) were added to the tube for fecal sample lysis. Tubes were incubated at 4°C for 30 min, vortexed for 1 min, and then spun at 16,000 × *g* for 1 min to pellet debris. 100 mL supernatant was added to one well of a 96-well white plate (Costar 3610) with two technique replicates for each sample, and then 100 mL of a 25:1 Nano-Glo Luciferase buffer to Nano-Glo Luciferase substrate mix was added to each well. The plate was incubated in the dark for 3 min at room temperature. Luminescence values were read on a Cytation 3 cell imaging multi-mode reader (BioTek).

### Fecal DNA extraction and quantification of oocysts using qPCR

DNA was extracted from fecal pellets using the QIAamp PowerFecal DNA kit (Qiagen) according to the manufacturer’s protocol. Oocyst numbers were quantified using qPCR with the *C. parvum* glyceraldehyde-3-phosphate dehydrogenase (GAPDH) primers (Table S2), as described previously (59). A standard curve was established by purifying genomic DNA from a known number of oocysts following a serial dilution. Reactions were performed on a QuantStudio 3 real-time PCR system (Thermo Fisher) with the amplification conditions as previously described (59).

### Genotyping of transgenic parasites

To check for the successful insertion of the target sequence into the genomic locus of specific gene, PCR was performed on 1 mL purified fecal DNA using Q5 Hot Start high-fidelity 2× master mix (New England Biolabs, #M0494S) with primers listed in Table S2. The primer sets for genotyping were designed with one primer located within the homology fragment and the other positioned outside of the insertion site. PCRs were performed on a Veriti 96-well thermal cycler (Applied Biosystems) with the following cycling conditions: 98°C for 30 s, followed by 35 cycles of 98°C for 15 s, 60°C for 30 s, and 72°C for 2 min, with a final extension of 72°C for 2 min. Melting temperature and extension time may vary from different PCR for the specific primers and distinct product length. PCR products were resolved on a 1.0% agarose gel containing GelRed (diluted 1:10,000; Biotium) and imaged on a ChemiDoc MP imaging system (Bio-Rad).

### RNA extraction and RT-qPCR

HCT-8 cells grown on coverslips with 80% confluency were infected with 1 × 10^5^ oocysts per well. RNA was collected at 24 hpi and extracted using the RNeasy minikit (Qiagen), treated with the RQ1 DNase Kit (Promega), and converted to cDNA using the SuperScript VILO cDNA synthesis kit (Thermo Fisher Scientific). Reverse transcription quantitative PCR (RT-qPCR) was performed using a QuantStudio 3 real-time PCR system (Thermo Fisher Scientific) with SYBR green JumpStart Taq ReadyMix (Sigma) using primers listed in Table S2. The following conditions were used for RT-qPCR: priming at 95°C for 2  min, followed by 40 cycles of denaturing at 95°C for 10 s, annealing at 60°C for 20 s, and extension at 72°C for 30 s, followed by a melt curve analysis to detect nonspecific amplification. Relative gene expression was calculated with the ΔΔCT method (60) using *C. parvum* GAPDH as the reference gene. All the primers are specified in Table S2.

### Indirect immunofluorescence microscopy

HCT-8 cells grown on coverslips with 80% confluency were infected with 1 × 10^5^ oocysts per well. Infected cells were fixed with 4% formaldehyde for 10 min and washed three times with PBS at specific timepoints post-infection. The fixed samples were then permeabilized and blocked with blocking buffer consisting of 1% BSA and 0.1% Triton X-100 (Sigma) in PBS. Parasites were co-stained with a polyclonal rabbit antibody to *C. parvum* called Pan Cp, the lectin VVL, or several mouse mAbs that we have previously characterized based on their reaction to different life cycle stages of *C. parvum* grown *in vitro* (31). Primary antibodies were diluted in blocking buffer: rat anti-HA (Thermo Fisher Scientific, #50-100-3325) was used at 1:500, rabbit anti-HA (Thermo Fisher Scientific, #71-5500) was used at 1:500 (for U-ExM), mAb 1B5 (hybridoma supernatant) was used at 1:250, mAb 1A5 was used at 1:500, mAb 4D8 was used at 1:50, VVL was used at 1:10,000, and Pan Cp (rabbit polyclonal antibody) was used at 1:10,000. Cells were incubated with primary antibodies for 1 h at room temperature, washed three times with PBS, and then incubated for 1 h at room temperature in secondary antibodies conjugated to Alexa Fluor dyes (Thermo Fisher Scientific) diluted 1:1,000 in blocking buffer. Nuclear DNA was stained with Hoechst (Thermo Fisher Scientific) diluted 1:2,000 in blocking buffer for 20 min at room temperature and then mounted with Prolong Glass Antifade Mountant (Thermo Fisher Scientific). Images were captured on a Zeiss Axioskop Mot Plus fluorescence microscope equipped with a 100×, 1.4 N.A. Zeiss Plan Apochromat oil objective lens or on a Zeiss LSM880 laser scanning confocal microscope equipped with a 63×, 1.4 N.A. Zeiss Plan Apochromat oil objective lens. Images were acquired using AxioVision Rel v 4.8, software, or ZEN v2.1, v2.5 software. Images were adjusted in ImageJ v2.0.0.

### Mitotracker staining

HCT-8 cells grown on coverslips with 80% confluency were infected with 1 × 10^5^ oocysts per well. For the co-staining assay, infected cells were stained with MitoTracker Red CMXRos (Thermo Fisher Scientific, #M7512; 50 nM final concentration) at 24 hpi for 45 min and washed three times with PBS. Treated cells were then fixed and stained with rat anti-HA at a 1:500 followed by anti-rat Alexa Fluor 488 (Thermo Fisher Scientific) at 1:1,000. Images were captured on a Zeiss LSM880 laser scanning confocal microscope equipped with a 63×, 1.4 N.A. Zeiss Plan Apochromat oil objective lens and acquired using ZEN v2.1, v2.5 software. For CCCP treatment, infected cells were washed twice with DPBS and treated with 10 µM carbonyl cyanide m-chlorophenyl hydrazone (CCCP, Sigma, #C2759) in 1% dimethyl sulfoxide (DMSO) media or 1% DMSO control for 2 h (from 22 to 24 hpi). MitoTracker Red CMXRos (50 nM final concentration) was added to the culture 45 min before 24 hpi and washed out using PBS 3 times. Treated cells were then fixed at 24 hpi with 4% formaldehyde and stained with mouse monoclonal antibody Cp 1A5, followed by anti-mouse Alexa Fluor 488 (Thermo Fisher Scientific). Images were captured on a Zeiss Axioskop Mot Plus fluorescence microscope equipped with a 100×, 1.4 N.A. Zeiss Plan Apochromat oil objective lens and acquired using AxioVision Rel v 4.8 software. Images were adjusted in ImageJ v2.0.0.

Quantification was performed using CellProfiler 4.2.8 (https://cellprofiler.org/). The pipeline included steps for parasite identification by antigen staining, subsequent mitosome identification, and measurement of the mitosome staining intensity. Parasites were stained using the *C. parvum* marker mAb Cp 1A5, and individual parasites were identified by setting the size of objects from 1.5 µm to 3.5 µm. Separately, individual MitoTracker-positive structures were identified based on a size window of 200 nm to 1 µm to identify the mitosome (the maximum size is larger than the actual predicted size of the mitosome due to the diffraction-limited nature of light microscopy and the scattered light emitted from small fluorescent objects). Integrated intensities of MitoTracker staining were collected from at least 150 parasites from two independent experiments. Images were analyzed from control and ∆*aox* KO parasites using identical parameters.

### Expansion microscopy

U-ExM was applied as described previously (61). HCT-8 cells were infected the same way as described for immunofluorescence staining. Samples were embedded overnight in a mixture of 1% acrylamide and 0.7% formaldehyde for protein anchor and crosslinking prevention. The formed gel was transferred into the denaturation buffer and denatured at 95°C. Polymerization of expansion gel was performed on ice containing monomer solution (19% sodium acrylate/10% acrylamide/0.1% (1,2-Dihydroxyethylene) bis-acrylamide), 0.5% ammonium persulfate, and 0.5% tetramethyl ethylenediamine. Polymerized gels were denatured at 95°C for 90 min in the denaturation buffer (200 mM SDS, 200 mM NaCl, 50 mM Tris-Base, pH = 9.0) and expanded in pure H_2_O overnight. The next day, the expansion ratio of fully expanded gels was determined by measuring the diameter of the gels. Well-expanded gels were shrunk in PBS and stained with primary (rabbit anti-HA at 1:200, rat Pan Cp at 1:500), secondary antibodies (Alexa Fluor dyes at 1:500), and Hoechst (at 1:500) diluted in freshly prepared PBS/BSA 2% at room temperature for 6 h. Three washes with PBS/0.1% Tween for 10 min were performed after each staining. Stained gels were expanded again in pure H2O overnight for further imaging. Images were captured on a Zeiss LSM880 laser scanning confocal microscope equipped with a 63×, 1.4 N.A. Zeiss Plan Apochromat oil objective lens and acquired using ZEN v2.1, v2.5 software.

### Transmission electron microscopy

Mouse intestinal spheroids were cultured on transwells to create the air-liquid interface culture using a modification of previously published methods (59). Cells were infected with WT sporozoites. Monolayers were fixed at intervals post-infection in a freshly prepared mixture of 1% glutaraldehyde (Polysciences Inc.) and 1% osmium tetroxide (Polysciences Inc.) in 50 mM phosphate buffer for 30 min at 4°C, then embedded in 3% agarose. Samples were rinsed in cold dH2O prior to staining with 1% aqueous uranyl acetate (Ted Pella Inc.) at 4°C for 3 h. After several rinses in dH2O, samples were dehydrated in a graded series of ethanol and embedded in Eponate 12 resin (Ted Pella Inc.). Sections of 95 nm were cut with a Leica Ultracut UCT ultramicrotome (Leica Microsystems Inc.) and stained with uranyl acetate and lead citrate. Sections were viewed on a JEOL 1200 EX transmission electron microscope (JEOL USA Inc.) equipped with an AMT 8 megapixel digital camera and AMT Image Capture Engine v602 software (Advanced Microscopy Techniques).

### *C. parvum* growth assay and drug treatment *in vitro*

HCT-8 cells were plated at 1 × 10^5^ cells per well in black-sided, optically clear-bottomed 96-well plates (Greiner Bio-One) and grown for 24 h until confluent. Cells were infected with 5 × 10^3^ bleached oocysts per well. After 24 h of infection/treatment, cells were fixed in 4% formaldehyde for 10 min, washed three times with PBS, and then permeabilized and blocked in PBS containing 0.1% Triton X-100 and 1% BSA for 20 min. *C. parvum* parasites were labeled with rabbit Pan Cp diluted 1:2,000 in blocking buffer, followed by Alexa Fluor goat anti-rabbit 488 secondary antibody (1:1,000). Host cell nuclei were stained with Hoechst for 20 min. Plates were imaged with a 10× objective on a BioTek Cytation 3 cell imager (20 images per well in a 5 × 4 grid). BioTek Gen5 software version 3.08 (Agilent) was used to quantify the total number of parasites (puncta in the GFP channel) and host cells (nuclei in the DAPI channel) per well.

For dose-response *C. parvum* growth inhibition assay of SHAM (Thermo Fisher Scientific, # AC132620050) and 8-HQ (Thermo Fisher Scientific, #AA4127218) for *C. parvum*, drugs were tested in a 9-point 1:2.5 serial dilution series starting at 6 μM (SHAM) and 1 μM (8-HQ). *C. parvum* growth assays were performed as described above. EC_50_ and EC_90_ values were calculated in GraphPad Prism 9 using a nonlinear regression curve fit with six replicates per data point (three technical replicates from two independent experiments).

### Phylogenetic analysis

All amino acid sequences were retrieved from the NCBI protein database. Multiple sequence alignment was performed using the L-INS-i algorithm in MAFFT (https://mafft.cbrc.jp/alignment/server/index.html) and visualized with ESPript (https://espript.ibcp.fr/ESPript/ESPript/index.php). Phylogenetic trees were constructed using the maximum-likelihood method with the JTT matrix-based model and 500 bootstrap replicates in MEGA (version 12.0.11). Tree visualization was carried out using iTOL (https://itol.embl.de). Conserved residues were identified using the NCBI Conserved Domain Database.

### Statistical analysis

All statistical analyses were performed in GraphPad Prism 10 (GraphPad Software) unless otherwise specified. Information on replicates, specific statistical tests, corrections for multiple comparisons, and *P* values are given in the figures or figure legends. *P* values of ≤ 0.05 were considered statistically significant.

## Data Availability

All of the data are found in the manuscript or supplemental material.

## SUPPLEMENTAL MATERIAL

Supplemental material mbio.01120-25-s0001.pdf Supplemental figures and tables.

ASM does not own the copyrights to Supplemental Material that may be linked to, or accessed through, an article. The authors have granted ASM a non-exclusive, world-wide license to publish the Supplemental Material files. Please contact the corresponding author directly for reuse.
