# Supplementary material for: Assessing the function of the alternative electron transport chain in the Cryptosporidium parvum mitosome
Source: mBio. 2025 Nov 13;16(12):e01120-25. doi: 10.1128/mbio.01120-25 (PMC12691609; doi:10.1128/mbio.01120-25)
Supplement: Supplemental material — Supplemental figures and tables. [file mbio.01120-25-s0001.pdf]

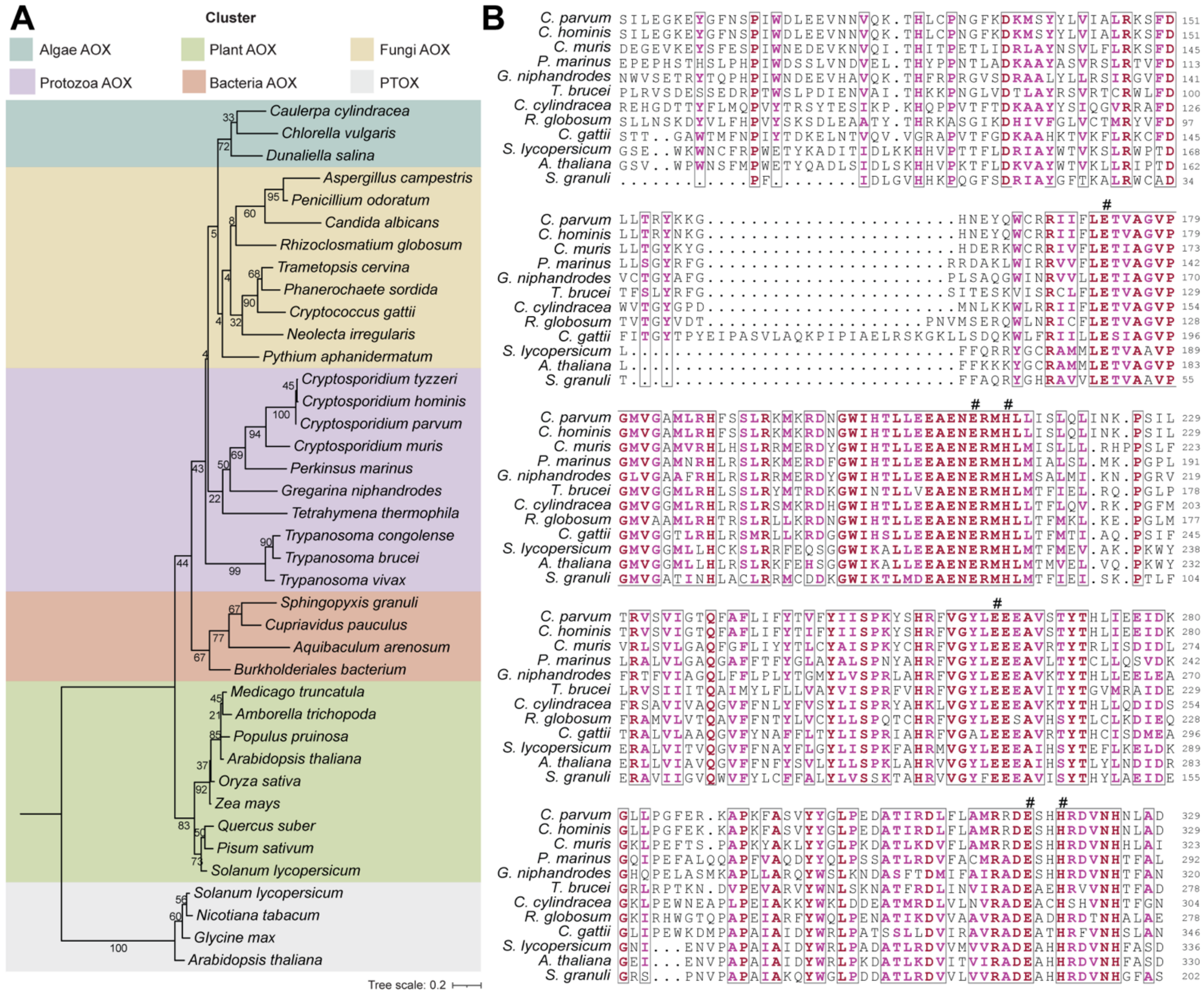

Figure S1 Phylogenetic analysis and multiple sequence alignment of CpAOX and its orthologs. (A). Maximum-likelihood phylogenetic tree showing the evolutionary relationships of CpAOX and AOX proteins across organisms in algae (cyan), fungi (yellow), bacteria (orange), plant (green), and other protozoa (purple). Plastid terminal oxidase (PTOX, grey) was used as out group. Bootstrap support values are shown as percentages next to the nodes (based on 500 replicates). Scale bar = 0.2 of substitutions per amino acid site. (B). Multiple sequence alignment of homologous region of CpAOX and its homologs from other organisms. Fully conserved residues are shown in red, residues with low variability in magenta, and highly variable residues in black. Conserved residues forming the diiron center within the AOX domain are indicated with hash marks (#). Protein sequences were aligned using MAFFT with the L-INS-i method.

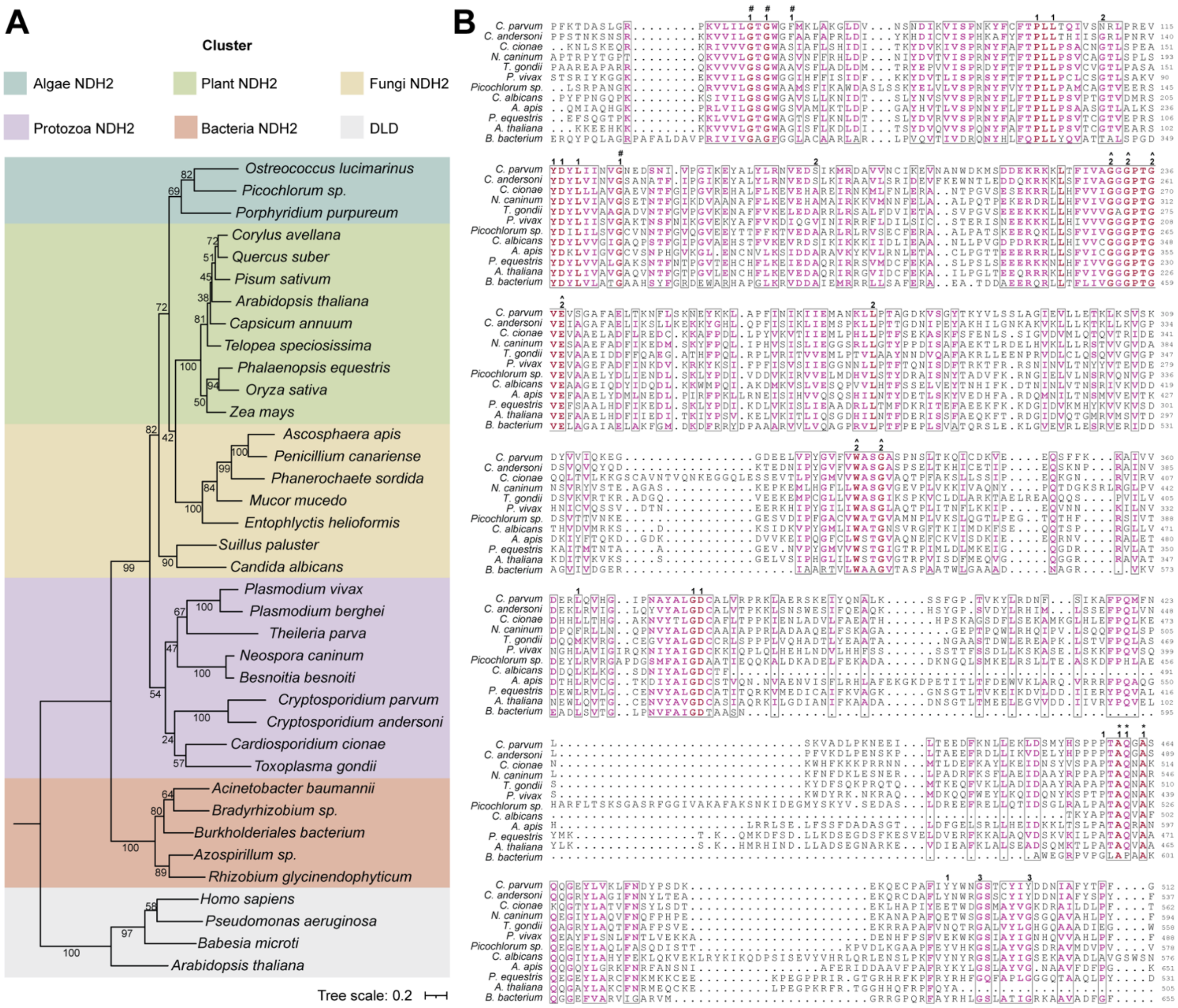

Figure S2 Phylogenetic analysis and multiple sequence alignment of CpNDH2 and its orthologs. (A). Maximum-likelihood phylogenetic tree showing the evolutionary relationships of CpNDH2 and NDH2 proteins across organisms in algae (cyan), fungi (yellow), bacteria (orange), plant (green), and other protozoa (purple). Dihydrolipoamide dehydrogenase (DLD, grey) was use as outgroup. Bootstrap support values are shown as percentages next to the nodes (based on 500 replicates). Scale bar = 0.2 of substitutions per amino acid site. (B). Multiple sequence alignment of homologous region of CpNDH2 and its homologs from other organisms. Fully conserved residues are shown in red, residues with low variability in magenta, and highly variable residues in black. Conserved residues in 1st Rossmann domain, 2nd Rossmann domain, and C terminus were marked as number 1, 2, and 3 respectively. Residues with proposed function was marked with hash (#) for

FAD stabilization, asterisk (*) for quinone binding site, and caret (^) for NAD(P)H stabilization. Protein sequences were aligned by MAFFT using L-INS-i method.

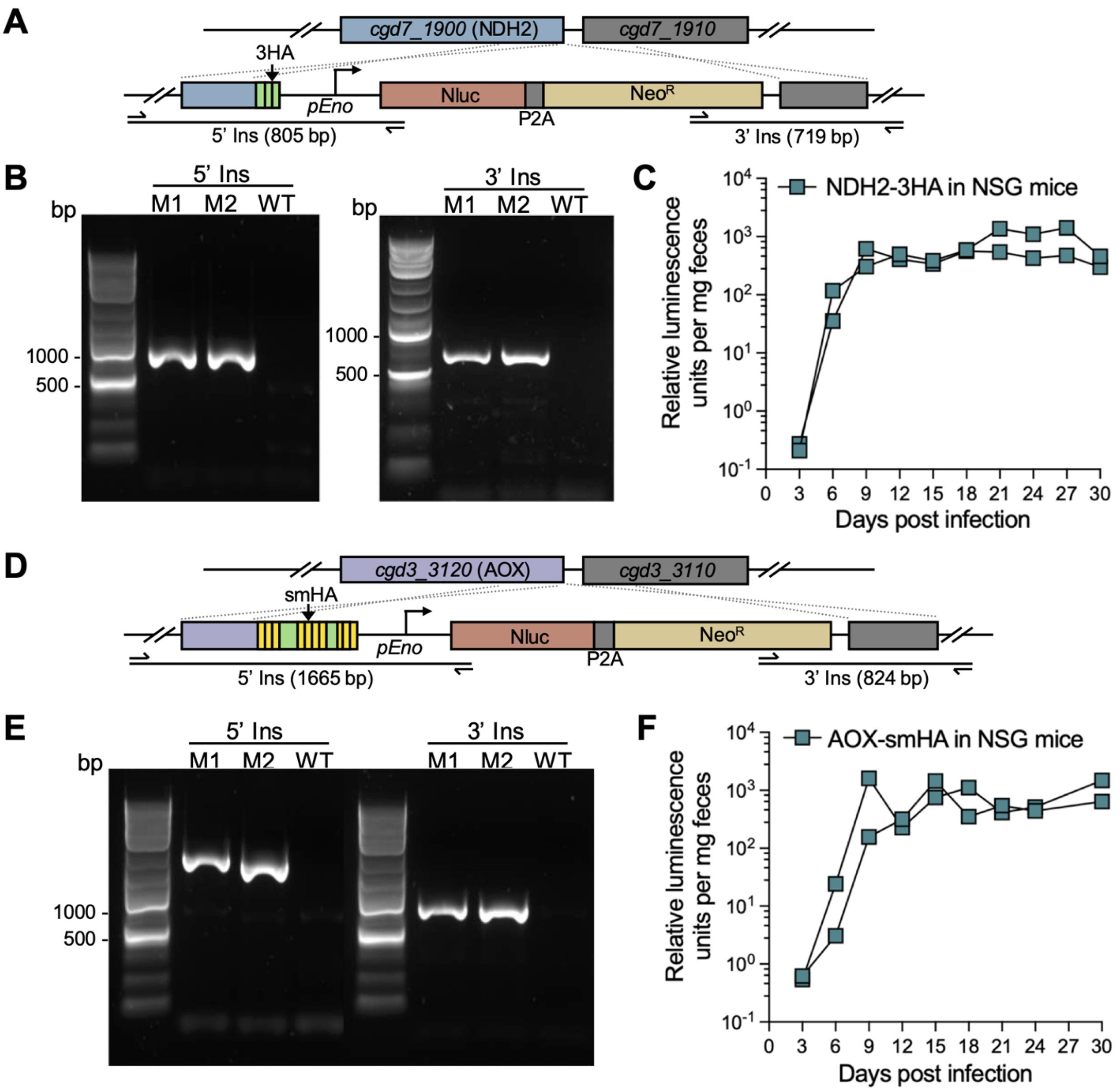

Figure S3 Genotype validation and growth assessment of tagging parasites. (A) Schematic of the NDH2-3HA-tagged endogenous locus in stable transgenic parasites. *C. parvum* sporozoites were co-transfected with NDH2-3HA-Nluc-P2A-NeoR tagging plasmid and CRISPR/Cas9 plasmid containing sgRNA specific to the C terminal of NDH2. Nluc, Nanoluc luciferase; P2A, split peptide; NeoR, neomycin resistant cassette. 5' Ins and 3' Ins refer to fragments used for diagnostic PCR in B. (B) Genotype analysis of NDH2-3HA-tagged *C. parvum* strain by PCR. M1 and M2, NDH2-3HA-tagged parasites from two NSG mice; WT, wild type parasite. The product 5' Ins is specific for the 5' CRISPR integration at the genomic locus of NDH2 with a forward primer located outside the

insertion site. The product 3' Ins is specific for the 3' CRISPR integration at the genomic locus of NDH2 with a reverse primer located outside the insertion. Primers are defined in Table S2. (C) Relative luminescence per milligram of feces from NSG mice challenged by NDH2-3HA-tagged parvum. Each data point represents a single fecal pellet, and each connecting line represents an individual infected NSG mouse. (D) Schematic of the AOX-smHA-tagged endogenous locus in stable transgenic parasites. 5' Ins and 3' Ins refer to fragments used for diagnostic PCR in E. (E) Genotype analysis of AOX-smHA-tagged *C. parvum* strain by PCR. M1 and M2, AOX-smHA-tagged parasites from two NSG mice. The product 5' Ins is specific for the 5' CRISPR integration at the genomic locus of AOX with a forward primer located outside the insertion site. The product 3' Ins is specific for the 3' CRISPR integration at the genomic locus of AOX with a reverse primer located outside the insertion. Primers are defined in Table S2. (F) Relative luminescence per milligram of feces from NSG mice challenged by AOX-smHA-tagged parvum. Each data point represents a single fecal pellet, and each connecting line represents an individual infected NSG mouse.

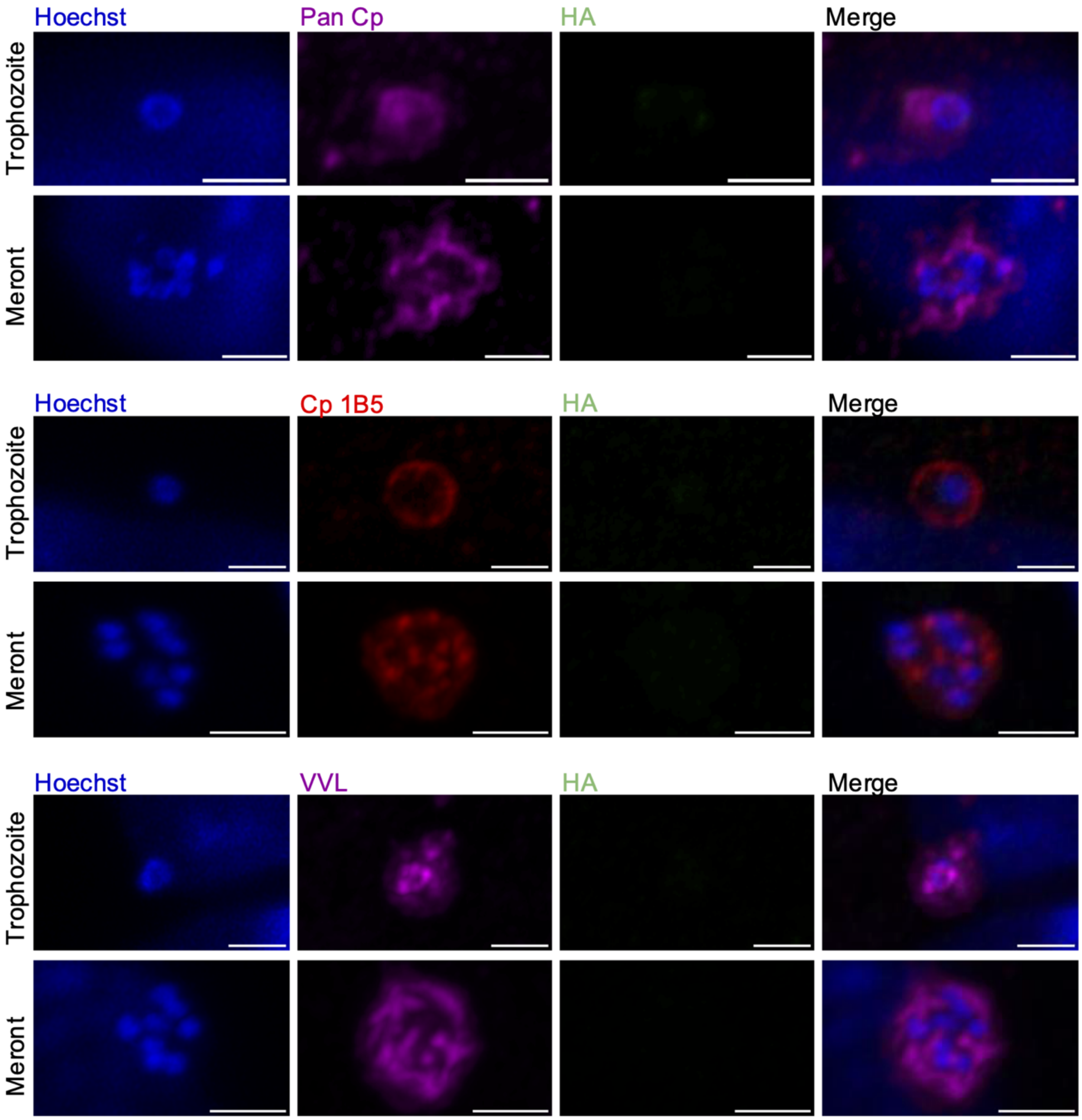

Figure S4 Validation of antibody specificity used in IFA for protein localization. HCT-8 cells were infected with wild-type oocysts. At 24 hpi, coverslips were fixed and stained with rat anti-HA followed by goat anti-rat IgG Alexa Fluor 488 (green), Pan Cp followed by goat anti-rabbit IgG Alexa Fluor 647 (magenta) or Cp 1B5 followed by goat anti-mouse IgG Alexa Fluor 568 (red) or VVL followed by streptavidin Alexa Fluor 647 (magenta), and Hoechst (blue) for nuclear staining. Scale bars, 2 μm.

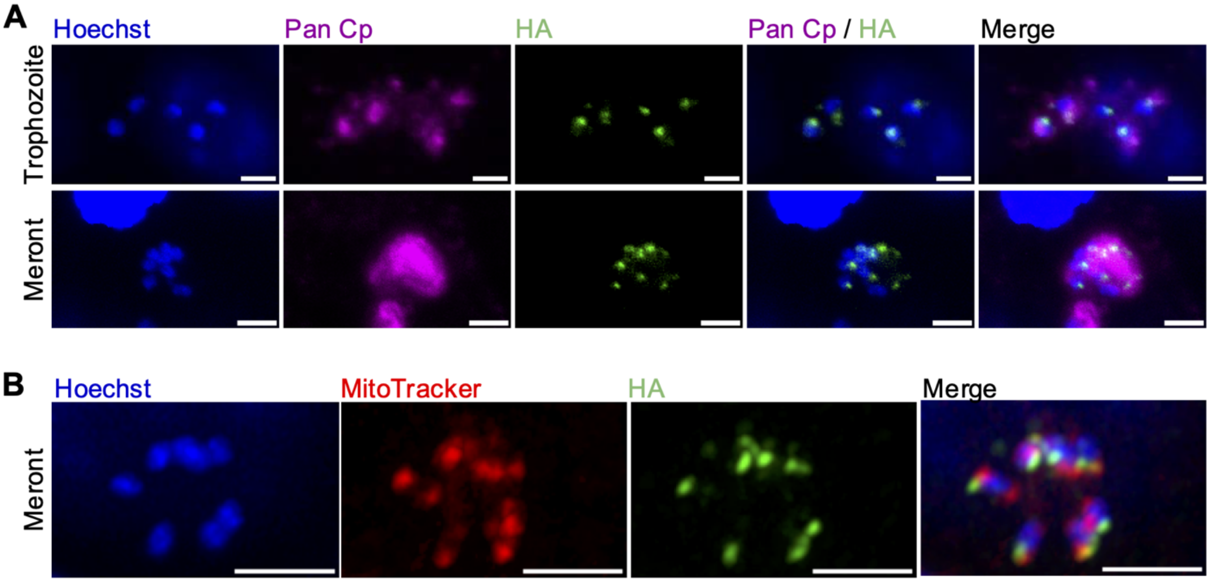

Figure S5 Localization of AOX in *C. parvum* using IFA. (A) Immunofluorescence staining of transgenic AOX-smHA-tagged parasites. HCT-8 cells were infected with AOX-smHA oocysts. At 24 hpi, coverslips were fixed and stained with rat anti-HA followed by goat anti-rat IgG Alexa Fluor 488 (green), Pan Cp followed by goat anti-rabbit IgG Alexa Fluor 647 (magenta), and Hoechst (blue) for nuclear staining. Scale bars, 2 μm. (B) Colocalization of AOX-smHA and MitoTracker. HCT-8 cells were infected with AOX-smHA oocysts. At 24 hpi, infected cells were stained with Mitotracker (50 nM) and then fixed. Fixed coverslips were stained with rat anti-HA followed by goat anti-rat IgG Alexa Fluor 488 (green), and Hoechst (blue) for nuclear staining. Scale bars, 2 μm.

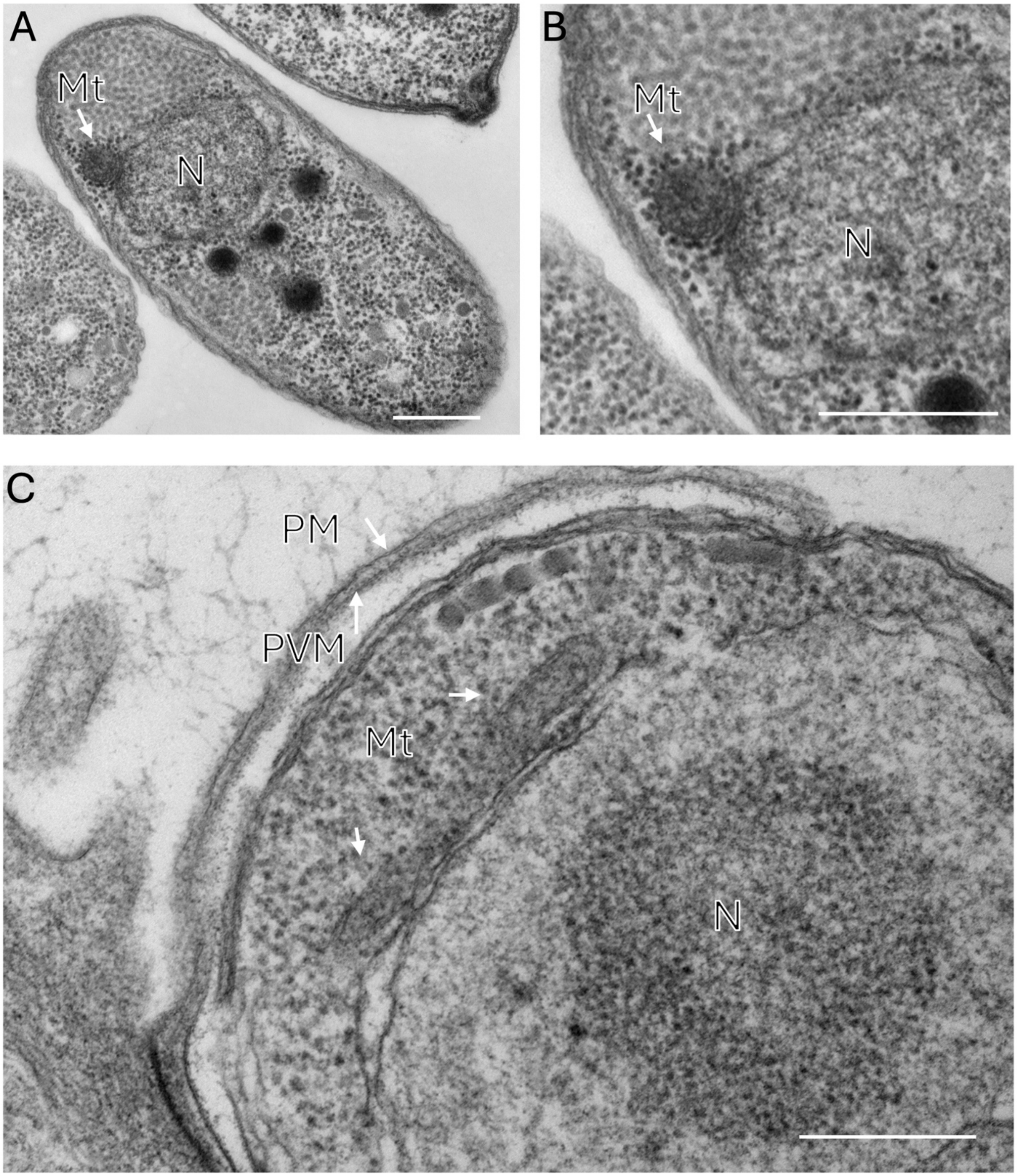

Figure S6 Transmission electron microscopy of the mitosome in *C. parvum*. A) Longitudinal section of sporozoite with posterior mitosome (Mt) adjacent to nucleus (N). B) Enlargement of A. C) Cross section of trophozoite showing elongate mitosome adjacent to nucleus. PM, plasma membrane of host cell, PVM, parasitophorous vacuole membrane. Scale bars 500 nm.

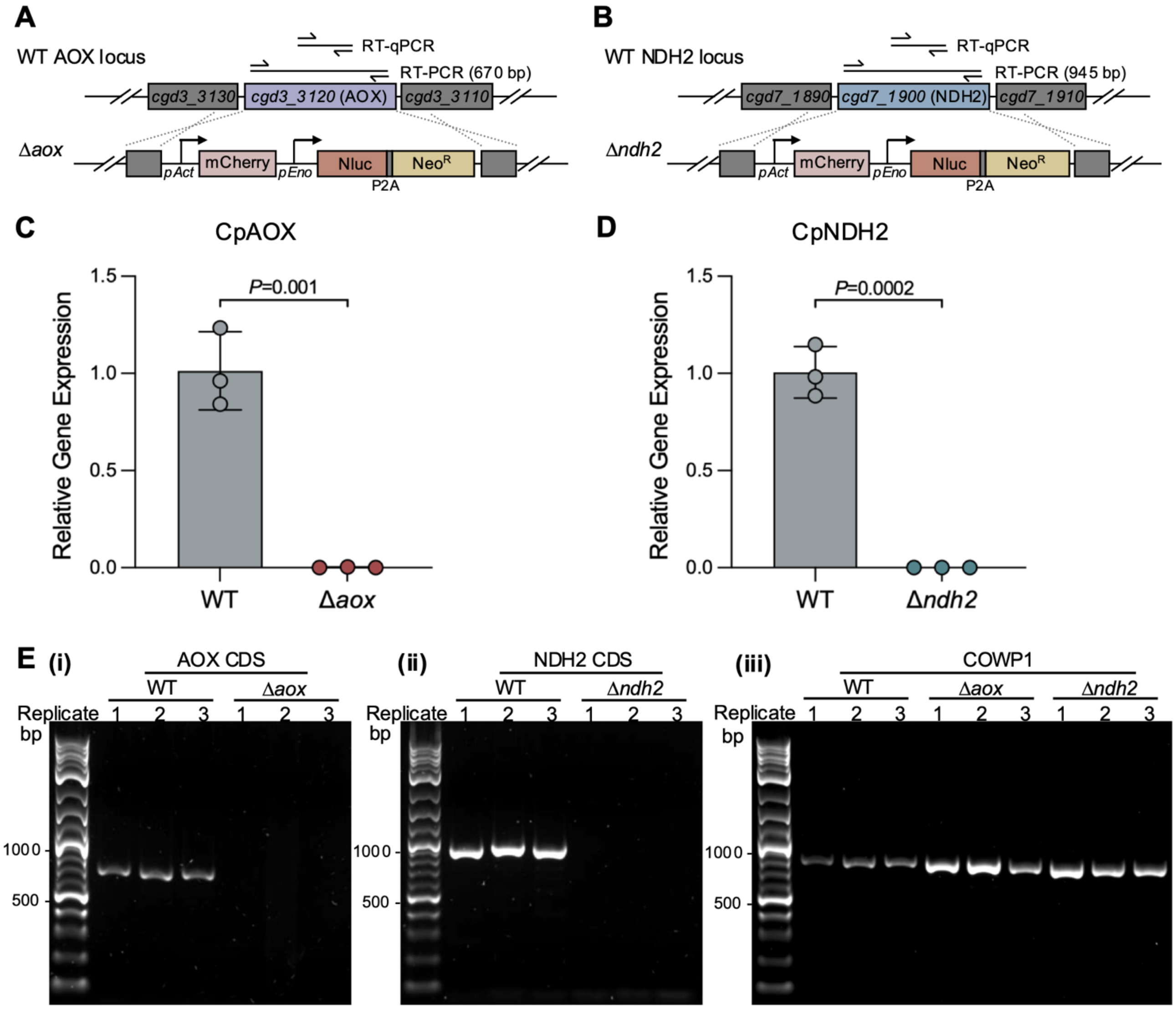

Figure S7 Testing the presence of AOX or NDH2 mRNA in respective knockout strains. Diagrams of the strategies to construct (A) Δ*aox* and (B) Δ*ndh2* transgenic parasites. Construct was designed to replace the gene locus with an mCherry and Nluc-P2A-NeoR cassette. The top line shows the genomic locus and the bottom line the successfully targeted transgenic locus. sgRNA, small guide RNA. qPCR and CDS PCR refer to fragments used for diagnostic qPCR and PCR in C, D, and E. Primers are specified in Table S2. (C) Relative transcription level of AOX gene in Δ*aox* parasites compared with that in WT parasites at 24hpi, as determined by RT-qPCR. (D) Relative transcription level of NDH2 gene in Δ*ndh2* parasites compared with that in WT parasites at 24hpi, as determined by RT-qPCR. HCT-8 cells were infected with *C. parvum* oocysts and RNA were collected at 24 hpi. Data from the Cryptosporidium GAPDH gene were used for data normalization.

Data were collected from 3 replicates for each strain. Statistical analysis was performed using the Student’s t test. (E) RT-PCR analysis of the coding sequence of either (i) AOX or (ii) NDH2 in the Δ*aox* or Δ*ndh2* parasites. (iii) *C. parvum* oocyst wall protein (COWP1) was used as the reference gene.

Table S1 BLASTP analyses of AOX and NDH2

Alternative oxidase (AOX)

C. parvum cgd3_3120 BLASTP against *nr* in NCBI (https://blast.ncbi.nlm.nih.gov/Blast.cgi)

Category	Organism	Protein ID	Annotation	E value
Top hit in *Cryptosporidium* spp.	*Cryptosporidium hominis*	XP_665993.1	alternative oxidase	0
Top hit in Apicomplexa (exclude Cryptosporidium spp.)	*Gregarina niphandrodes*	XP_011134197.1	alternative oxidase	2E-77
Top hit in Parasites (exclude Apicomplexa)	*Perkinsus marinus*	XP_002788619.1	alternative oxidase	1E-83
Top hit in Eukaryotes (exclude parasites)	*Caulerpa cylindracea*	AXB99490.1	mitochondrial alternative oxidase	3E-73

Reciprocal BLASTP against *C. parvum* in CryptoDB (https://cryptodb.org/cryptodb/app)

Organism	Protein ID	Annotation	Top 3 hits in *C. parvum*	Score	E value
Cryptosporidium hominis	XP_665993.1	alternative oxidase	*cgd3_3120*	677	0
			cgd8_2000	28.9	0.95
			cgd8_1410	26.2	5.6
Gregarina niphandrodes	XP_011134197.1	alternative oxidase	*cgd3_3120*	250	3e-82
			cgd7_3873	27.7	0.66
			cgd7_1470	26.9	3.8
Perkinsus marinus	XP_002788619.1	alternative oxidase	*cgd3_3120*	265	2e-88
			cgd8_480	26.6	5.4
Caulerpa cylindracea	AXB99490.1	mitochondrial alternative oxidase	*cgd3_3120*	239	4e-78
			cgd7_2620	28.9	0.93
			cgd4_2900	26.9	4.8

Type II NADH dehydrogenase (NDH2)

C. parvum cgd7_1900 BLASTP against *nr* in NCBI (https://blast.ncbi.nlm.nih.gov/Blast.cgi)

Category	Organism	Protein ID	Annotation	E value
Top hit in *Cryptosporidium* spp.	*Cryptosporidium andersoni*	XP_067067602.1	NADH dehydrogenase	0
Top hit in Apicomplexa (exclude Cryptosporidium spp.)	*Cardiosporidium cionae*	KAF8820016.1	naDH dehydrogenase (NDH2-II)	1E-87
Top hit in Plants	*Phalaenopsis equestris*	XP_020589374.1	external alternative NAD(P)H-ubiquinone oxidoreductase	1E-63
Top hit in Fungi	*Ascosphaera apis*	KZZ93509.1	external NADH-ubiquinone oxidoreductase 2	7E-53

Reciprocal BlASTP against *C. parvum* in CryptoDB (https://cryptodb.org/cryptodb/app)

Organism	Protein ID	Annotation	Top 3 hits in *C. parvum*	Score	E value
Cryptosporidium andersoni	XP_067067602.1	NADH dehydrogenase	*cgd7_1900*	576.0	0
			cgd6_1410	35.8	0.017
			cgd7_4333	30.8	0.49
Cardiosporidium cionae	KAF8820016.1	naDH dehydrogenase (NDH2-II)	*cgd7_1900*	291.0	2e-91
			cgd1_1920	30.8	0.59
			cgd8_1100	29.6	1.4
Phalaenopsis equestris	XP_020589374.1	external alternative NAD(P)H-ubiquinone oxidoreductase	*cgd7_1900*	220	6e-65
			cgd8_1340	28.1	3.9
			cgd4_2300	27.3	5.6
Ascosphaera apis	KZZ93509.1	external NADH-ubiquinone oxidoreductase 2	*cgd7_1900*	191	2e-53
			cgd8_1280	30.4	0.37
			cgd5_730	30.4	0.84

Table S2 Primers used in the study.

Category	Usage	Fragment	Template	Forward primer (5' → 3')	Reverse primer (5' → 3')
Plasmid construction	PCR	EnoP-Nluc-P2A-Neo^R	pCpGT1-3HA	ATGCATCTTCATTTAGTATCTTAGGTCG	AATTAAGATAAAAGAAAAACTTAATCGATAC
	PCR	pUC19 backbone	pCpGT1-3HA	GGGGATCCTCTAGAGTCGAC	GGGTACCGAGCTCGAATT
	PCR	smHA cassette / 3HA cassette	pCpGT1-3HA/ pCpGT2-smHA	GCTAGCAAGGGCTCGGGC	GGCGCGCCAAATAAAGTAAAGTTTATCG
	PCR	pCRISPR/Cas9 backbone	pCpGT1-3HA	GTTTTAGAGCTAGAAATAGCAAG	CCCAACACTTAACCTTTCAGT
	PCR	AOX-5'homology arm	*C. parvum* genomic DNA	GAATTCGAGCTCGGTACCCTTACTCTACTTGAAGAGGCTGAA	GAGCCCGAGCCCTTGCTAGCTTCTCCATTTAATCTTATATCTGCG
	PCR	AOX-3'homology arm	*C. parvum* genomic DNA	TTTTTCTTTTTATCTTAATTTAATTTAGATATTTATTGAAATATTTTATTTCATTC	GTCGACTCTAGAGGATCCCCGTCTCATCATCAATTACAACAAG
	Cloning	AOX-PAM mutation	-	AGAGAAAAGCTCCGAAATTTGC	CAAATCCTGGAAGAAGACC
	Cloning	AOX-sgRNA1-Linker	-	CTGAAAGGTTAAGTGTTGGGAGTAGACGGAGGCAAATTTCGTTTTAGAGCTAGAAATAGC	
	PCR	NDH2-5'homology arm	*C. parvum* genomic DNA	TGAATTCGAGCTCGGTACCCTCTCACGGAAGAAGACTTC	GAGCCCGAGCCCTTGCTAGCGTGAGAAACGTTCATTTTGTAG
	PCR	NDH2-3'homology arm	*C. parvum* genomic DNA	TTTTTCTTTTTATCTTAATTAAAGAAGAGATTTGACTATTTTTTG	GTCGACTCTAGAGGATCCCCCTGTTAATGGACTTTTGGC
	Cloning	NDH2-PAM mutation	-	CTACTTTGAATCCAAGTTCAAG	TATATGCTAATCGCCAGATATAC
	Cloning	NDH2-sgRNA1-linker	-	CTGAAAGGTTAAGTGTTGGGAACTGCGGGATCTTGAACTTGTTTTAGAGCTAGAAATAGC	
	PCR	AOX 5'UTR-mCherry-Nluc-P2A-Neo^R-AOX 3'UTR	pINS1-mCherry-Nluc-P2A-neo-INS1	TACAGCGGAATCTTATTTACAATCGTATTTTTTTTTAAATAATATTAATGCTCAGAATGAGTTGGTTATAAAC	AACTAAATTATTTTGAATGAAATAAAATATTTCAATAAATATCTAAATTAGCTTAATTAATCAGAAGAATTCGTC
	PCR	NDH2 5'UTR-mCherry-Nluc-P2A-Neo^R-NDH2 3'UTR	pINS1-mCherry-Nluc-P2A-neo-INS1	ATTAGAAATTAATTTCAAAAAAGAAATTCTTAGTAAATAAACTTTAAATTGCTCAGAATGAGTTGGTTATAAAC	GGCTAAATAAGATGAATTGTACACACAAAAAAATAGTCAAATCTCTTCTTTGCTTAATTAATCAGAAGAATTCGTC

Genotyping of transgenic strains	PCR	AOX-smHA 5’ Ins	*C. parvum* genomic DNA	GGTTGGTGCAATGCTTAGAC	GTGTGTGTGAAAAGCTGTC
	PCR	AOX-smHA 3’ Ins	*C. parvum* genomic DNA	GCTGAAGAACTTGGTGGTGA	GCCAATGCCGTCGTAAATAG
	PCR	NDH2-3HA 5’ Ins	*C. parvum* genomic DNA	GTTAGACCACGAAAGTTAGCAG	TGTGTGTGAAAAGCTGTC
	PCR	NDH2-3HA 3’ Ins	*C. parvum* genomic DNA	GCTGAAGAACTTGGTGGTGA	ACTATTGATCCAATGACTCTCTC
	PCR	AOX-KO 5’ Ins	*C. parvum* genomic DNA	CCAGCCATTGGAATTATGTCC	GTCATTTTTATAGCCCTAACGC
	PCR	AOX-KO 3’ Ins	*C. parvum* genomic DNA	GCTGAAGAACTTGGTGGTGA	GCTTATTATTACTTCGCCAATGC
	PCR	AOX-KO CDS	*C. parvum* genomic DNA/ RNA-RT cDNA	AGGCATCTTTGGCTACTCTCT	AGAAGGCAAACTGAGTCCCA
	PCR	NDH2-KO 5’ Ins	*C. parvum* genomic DNA	CTGCTGGAGTTTCCTCAATTAC	GTCATTTTTATAGCCCTAACGC
	PCR	NDH2-KO 3’ Ins	*C. parvum* genomic DNA	GCTGAAGAACTTGGTGGTGA	CGTAGAGTCAACAGTCAATGG
	PCR	NDH2-KO CDS	*C. parvum* genomic DNA/ RNA-RT cDNA	GACGGCCAAAGGTTCTCATCT	CGCAATCTCCAAGTGCGTATG
Oocyst quantification	qPCR	CpGAPDH	*C. parvum* genomic DNA	GAAGATGCGCTGGGAACAAC	CGGATGGCCATACCTGTGAG
mRNA validation	PCR	COWP1	RNA-RT cDNA	ACCGCTTCTCAACAACCATCTTGTCCTC	CGCACCTGTTCCCACTCAATGTAAACCC
	qPCR	CpAOX	RNA-RT cDNA	AATCAAACCATGCGGCTTCC	TTGCACCAACCATACCTGGG
	qPCR	CpNDH2	RNA-RT cDNA	TGGGACAAAATGAGCGACGA	GCAAGACCTCTATTCCTGCCA

Table S3 Plasmids used in the study

Plasmid Full Name	Fragments used	Usage
pNDH2-3HA-Nluc-P2A-Neo^R	pUC19 backbone	NDH2-3HA-tagging repairing plasmid
	NDH2-5'homology arm	
	3HA cassette	
	EnoP-Nluc-P2A-Neo^R	
	NDH2-3'homology arm	
pAOX-smHA-Nluc-P2A-Neo^R	pUC19 backbone	AOX-smHA-tagging repairing plasmid
	AOX-5'homology arm	
	smHA cassette	
	EnoP-Nluc-P2A-Neo^R	
	NDH2-3'homology arm	
AOX: mCherry-Nluc-P2A-Neo^R-AOX	AOX 5'UTR-mCherry-Nluc-P2A-Neo^R-AOX 3'UTR	AOX-Knockout repairing template (PCR product)
NDH2: mCherry-Nluc-P2A-Neo^R-NDH2	NDH2 5'UTR-mCherry-Nluc-P2A-Neo^R-NDH2 3'UTR	NDH2-Knockout repairing template (PCR product)
pCRISPR/Cas9-AOX-sgRNA1	pCRISPR/Cas9 backbone	CRISPR/Cas9 plasmid containing sgRNA specific to AOX
	AOX-sgRNA1-Linker	
pCRISPR/Cas9-NDH2-sgRNA1	pCRISPR/Cas9 backbone	CRISPR/Cas9 plasmid containing sgRNA specific to NDH2
	NDH2-sgRNA1-Linker	

Table S4 Transgenic *C. parvum* strains

Name	Plasmids used for co-transfection	Usage
NDH2-3HA	pNDH2-3HA-Nluc-P2A-Neo^R	NDH2-3HA-tagging
	pCRISPR/Cas9-NDH2-sgRNA1	
AOX-smHA	pAOX-smHA-Nluc-P2A-Neo^R	AOX-smHA-tagging
	pCRISPR/Cas9-AOX-sgRNA1	
Δ*aox*	AOX-mCherry-Nluc-P2A-Neo^R-AOX	*aox* knockout
	pCRISPR/Cas9-AOX-sgRNA1	
Δ*ndh2*	NDH2-mCherry-Nluc-P2A-Neo^R-NDH2	*Ndh2* knockout
	pCRISPR/Cas9-NDH2-sgRNA1	
